# Cognitive Biases and Moral-Regulatory Mechanisms in White-Collar Crime: A Systematic Review and Bibliometric Mapping of Psychological Pathways to Corporate and Financial Misconduct

**DOI:** 10.3390/bs16101915

**Published:** 2026-10-15

**Authors:** Jairo Dote-Pardo, Pedro Severino-González, Claudia Quezada-Baier, Nelson León-Silva, María Teresa Espinosa-Jaramillo

**Affiliations:** 1 Departamento de Ciencias Económicas y Administrativas, Facultad de Ciencias Jurídicas, Económicas y Administrativas, Universidad Católica de Temuco, Temuco 4810399, Chile; nleon2022@alu.uct.cl; 2 Centro de Gestión y Economía Aplicada (CGEA), Facultad de Ciencias Jurídicas, Económicas y Administrativas, Universidad Católica de Temuco, Temuco 4810399, Chile; 3 Departamento de Economía y Administración, Facultad de Ciencias Sociales y Económicas, Universidad Católica del Maule, Talca 3460000, Chile; pseverino@ucm.cl; 4 Departamento de Ciencias Económicas, Administrativas y de Comercio, Universidad de las Fuerzas Armadas ESPE, Sangolquí 171103, Ecuador; mtespinosa@espe.edu.ec

**Keywords:** white-collar crime, cognitive biases, moral disengagement, fraud triangle, corporate misconduct, systematic review, criminal psychology

## Abstract

**Background:** White-collar crime causes major economic and social harm, yet research on its psychological mechanisms remains fragmented across criminology, behavioral ethics, accounting and finance. **Objective:** To synthesize research on cognitive biases and moral-regulatory mechanisms underlying white-collar, corporate and financial misconduct, and to examine how they interact with organizational factors. **Methods:** A non-experimental, retrospective documentary design was used: a PRISMA 2020 systematic review with bibliometric mapping of Web of Science Core Collection and Scopus records (searches updated to October 2026; 107 studies included, 1975–2026). New records were screened independently by two reviewers, and study quality was appraised with the MMAT and JBI tools. **Results:** Moral self-regulation failures (rationalization, neutralization, moral disengagement) and dispositional traits (psychopathy, narcissism, the dark triad) are the most frequently examined mechanisms, followed by motivational pressures and risk-perception distortions such as overconfidence. These mechanisms are amplified by weak governance and attenuated by strong monitoring. Keyword co-occurrence analysis reveals three weakly connected thematic clusters. **Conclusions:** Cognitive distortions act as psychological catalysts within the pressure, opportunity and rationalization dimensions of the Fraud Triangle. The findings support bias-specific prevention: structural checks on executive overconfidence, ethics programs that target neutralization, and cultural interventions that disrupt normalized deviance.

## 1. Introduction

### 1.1. General Background

White-collar crime remains one of the most consequential yet least visibly disruptive forms of deviance in contemporary societies (Gottschalk, 2017). Since the seminal work of Edwin H. Sutherland (Sutherland, 1949), the concept has evolved to encompass a wide spectrum of non-violent offenses committed in occupational and organizational contexts, including corporate fraud, financial misconduct, market manipulation, bribery, and environmental violations (Simpson, 2019). Unlike conventional street crime, white-collar crime often occurs within legitimate organizational structures, is embedded in complex decision-making processes, and may be rationalized as routine business practice (Benson & Simpson, 2014). Its economic and social costs, however, are profound, undermining public trust, destabilizing markets, and exacerbating inequality (Clarkson & Darjee, 2022).

Despite decades of criminological and regulatory research, a persistent gap remains in understanding the psychological mechanisms that underlie white-collar offending (Ling et al., 2019). Traditional explanations have focused on structural pressures (e.g., strain, organizational culture, regulatory environments) or deterrence-based models rooted in rational choice theory (Parameswaran & Shahzad Shafi, 2025). While these frameworks illuminate contextual drivers, they often assume relatively stable, deliberative decision-making processes. Emerging evidence from cognitive psychology and behavioral economics challenges this assumption, demonstrating that human judgment is systematically shaped by heuristics and biases (Cati, 2025). Foundational work by Daniel Kahneman and Amos Tversky established that decision-making under uncertainty is frequently guided by cognitive shortcuts rather than fully rational calculations (Tversky & Kahneman, 1974; Kahneman & Tversky, 1979, 1984).

Within corporate and financial contexts, such cognitive distortions may play a pivotal role in enabling misconduct (La Rocca et al., 2025). Overconfidence bias can lead executives to underestimate legal and reputational risks (Hu et al., 2024). Self-serving bias may distort interpretations of ambiguous financial data in favor of personal or organizational gain (Amasino et al., 2023). Framing effects and bounded rationality may shape how ethical dilemmas are perceived, while motivated reasoning can facilitate the reinterpretation of questionable actions as acceptable (Leisge et al., 2025). Moreover, mechanisms such as moral disengagement and ethical fading allow individuals to suppress or neutralize moral self-sanctions, transforming potentially criminal conduct into cognitively manageable decisions (Bandura, 1999). These processes do not necessarily reflect overt criminal intent; rather, they may represent gradual psychological shifts in perception, justification, and risk assessment.

Recent high-profile corporate scandals have underscored how misconduct often emerges from incremental decisions made by otherwise socially integrated professionals (Eugster et al., 2024). Such patterns suggest that white-collar crime may be less about inherently “criminal personalities” and more about predictable cognitive vulnerabilities operating within permissive organizational environments. In this regard, integrating cognitive bias theory with criminological perspectives offers a promising avenue for advancing both theoretical clarity and preventive strategies.

Although individual studies have examined specific psychological constructs, such as overconfidence in financial misreporting (Schrand & Zechman, 2012), rationalization in occupational fraud (Shepherd & Button, 2019), or risk perception in regulatory compliance (Tong & Crosno, 2021), the literature remains fragmented. There is currently no comprehensive synthesis that systematically maps the range of cognitive biases implicated in white-collar crime and clarifies their mechanisms of action across corporate and financial settings. This fragmentation limits cumulative knowledge development and hinders the design of psychologically informed compliance and governance interventions.

To address this gap, the present study conducts a systematic review of empirical and conceptual research examining cognitive biases and related psychological mechanisms in white-collar, corporate, financial, occupational, and organizational crime. By synthesizing findings across disciplines—including criminal psychology, behavioral economics, organizational behavior, and corporate governance—this review seeks to: (i) Identify the most frequently examined cognitive biases associated with corporate and financial misconduct; (ii) Analyze how these biases interact with organizational and situational factors; (iii) Clarify theoretical pathways linking cognitive distortions to criminal or unethical outcomes; and (iv) Highlight implications for prevention, compliance design, and future research within criminal psychology.

It is important to clarify the conceptual boundaries of this review at the outset. Cognitive biases, in the strict sense established by Tversky and Kahneman (1974), are systematic deviations in judgment that arise from heuristic information processing rather than from motivational or social forces. The present review, however, also considers a set of related psychological mechanisms—including rationalization, moral disengagement, ethical fading, and normalization of deviance—that do not meet this narrow cognitive-heuristic definition but are theoretically and empirically inseparable from it in the white-collar crime literature.

These mechanisms function as the moral-regulatory pathways through which cognitive distortions are translated into permissible action, as established in the criminological and organizational-behavior literatures on techniques of neutralization, normalization of corruption, and moral disengagement (Sykes & Matza, 1957; Bandura, 1999; Ashforth & Anand, 2003; Bazerman & Tenbrunsel, 2011; Tenbrunsel & Messick, 2004). Accordingly, this review adopts a dual-construct scope—cognitive biases proper and their proximal moral-regulatory mechanisms—and reports both categories separately throughout the coding, tables, and synthesis rather than treating them as interchangeable. This distinction directly addresses a limitation of prior reviews. Acciarini et al. (2021), in a systematic review published in this journal, mapped cognitive biases in managerial strategic decision-making broadly but did not examine their operation within a criminal or misconduct-specific context, nor did it integrate criminological frameworks such as the Fraud Triangle or neutralization theory.

The present study extends this stream of research by narrowing the analytical lens to white-collar, corporate, and financial misconduct specifically, and by explicitly theorizing the link between cognitive-heuristic distortions and the moral-disengagement mechanisms that criminological research has long identified as necessary conditions for the commission of unethical or illegal acts (Coleman, 1987; Kish-Gephart et al., 2010).

### 1.2. Specific Background, Research Problem, and Review Questions

The specific background of the problem lies at the intersection of three bodies of evidence that have developed largely in parallel. First, archival research in accounting and finance consistently associates executive overconfidence with financial misreporting and fraud (Schrand & Zechman, 2012; Cao et al., 2024), but usually infers the bias from behavioral proxies rather than measuring it directly. Second, experimental and qualitative work in behavioral ethics shows that ordinary people behave dishonestly when they can preserve a positive self-concept (Mazar et al., 2008) and that ethical fading and neutralization reduce the moral salience of misconduct (Tenbrunsel & Messick, 2004; Shepherd & Button, 2019), but this evidence rarely examines actual corporate offenders. Third, criminological and organizational accounts emphasize opportunity structures and organizational enablers (Coleman, 1987; Greve et al., 2010; Palmer, 2012), with limited attention to the cognitive processes through which individuals interpret those opportunities. The result is a set of partly competing explanations: misconduct as the product of a miscalibrated but essentially rational calculus, as a gradual erosion of moral self-regulation, or as a structural outcome largely independent of individual cognition.

A further unresolved issue concerns the relationship between situational cognitive distortions and stable dispositional profiles. Research on the dark triad (Paulhus & Williams, 2002) and on corporate psychopathy (Babiak et al., 2010; Boddy, 2011) shows that individuals with psychopathic traits can reach managerial positions and are associated with workplace bullying, unfair supervision and counterproductive behavior; workplace harassment, or mobbing, has itself been characterized as a systematic pattern of hostile behavior directed at targeted employees (Leymann, 1996). Similarly, the Type A behavior pattern—characterized by competitiveness, time urgency and hostility (Friedman & Rosenman, 1974)—shares behavioral features with the performance-pressure profile often described in fraud research. Because individuals exhibiting biases such as overconfidence or moral disengagement may display behavioral repertoires similar to those of these profiles, it remains unclear whether cognitive biases and dispositional traits are interacting variables or independent explanatory pathways. Egan et al. (2015), for instance, report associations between moral disengagement and dark triad traits, suggesting that some moral-regulatory mechanisms may be partly rooted in personality.

These gaps and contradictions define the research problem addressed here. Without an integrative synthesis, it is not possible to determine which cognitive and moral-regulatory mechanisms are most consistently implicated in white-collar misconduct, how they combine with organizational conditions, and whether they constitute a distinct explanatory layer relative to structural and dispositional accounts. As a systematic review, this study does not test statistical hypotheses. It is instead guided by a theoretical working proposition derived from the evidence above: cognitive biases and moral-regulatory mechanisms function as proximal psychological pathways through which pressure, opportunity and rationalization are translated into misconduct, and their behavioral expression depends on organizational context. Accordingly, the four objectives stated above are operationalized as the following review questions (RQs):

RQ1. Which cognitive biases and moral-regulatory mechanisms are most frequently examined in relation to white-collar, corporate and financial misconduct?

RQ2. How do these mechanisms interact with organizational and situational factors?

RQ3. Through which theoretical pathways are they linked to criminal or unethical outcomes, and how do these pathways map onto the Fraud Triangle and its extensions?

RQ4. What implications follow for prevention, compliance design and future research?

## 2. Materials and Methods

### 2.1. Research Design

This study adopts a non-experimental, retrospective documentary (secondary-data) research design, implemented as a systematic literature review with bibliometric mapping, to synthesize empirical and conceptual research examining cognitive biases and related psychological mechanisms in white-collar, corporate, financial, occupational, and organizational crime. The review was designed to identify, categorize, and integrate findings across disciplines, including criminal psychology, behavioral economics, corporate governance, accounting, and organizational behavior. The procedures through which this design was implemented—search, screening, data extraction, quality appraisal and analysis—are described in Section 2.2, Section 2.3, Section 2.4, Section 2.5 and Section 2.6.

A systematic approach was adopted to ensure transparency, replicability, and methodological rigor. The review process followed established guidelines for systematic reviews in social sciences, incorporating structured search procedures, explicit inclusion and exclusion criteria, and standardized data extraction.

This review was conducted and is reported in accordance with the PRISMA 2020 statement (Page et al., 2021); the completed PRISMA 2020 checklist is provided as Supplementary Material.

This review was retrospectively registered on protocols.io (DOI: https://doi.org/10.17504/protocols.io.5qpvoj7bbg4o/v2). This version of the registration was completed after the original search (5 May 2026), study screening and data extraction had been performed; it is reported to enhance transparency and should not be interpreted as a prospective protocol. Following peer review, the updated and supplementary searches, the independent double screening of new records and the quality appraisal described below were registered as a protocol amendment (version 3; DOI: https://doi.org/10.17504/protocols.io.5qpvoj7bbg4o/v3) on 1 October 2026, before these procedures were executed. The implications of the retrospective registration of the original stages are discussed in Section 2.7.

The design of this review is grounded in three complementary theoretical traditions that have historically explained white-collar and organizational misconduct. The first is the criminological tradition originating in Sutherland’s (1949) concept of white-collar crime and elaborated through rational-choice and situational accounts of offender decision-making (R. Clarke & Cornish, 1985; R. V. Clarke, 1997), which frames misconduct as the outcome of a cost–benefit calculus rather than of a stable criminal disposition. The second is the neutralization and moral-disengagement tradition, beginning with Cressey’s (1953) classic analysis of embezzlement and Sykes and Matza’s (1957) techniques of neutralization, and extended by Ashforth and Anand’s (2003) on the normalization of corruption within organizations, Bandura (1999) on moral disengagement, and Tenbrunsel and Messick (2004) and Tenbrunsel and Smith-Crowe (2008) on ethical fading and the broader landscape of behavioral ethics research. This tradition explains how individuals reconcile unethical conduct with a positive self-concept, a process further documented by Mazar et al. (2008) and Bazerman and Tenbrunsel (2011). The third is the organizational-wrongdoing tradition, which situates individual cognitive and moral processes within permissive organizational and institutional contexts (Coleman, 1987; Vaughan, 1997; Pinto et al., 2008; Greve et al., 2010; Palmer, 2012) and identifies organizational, industry, and institutional-level enablers of misconduct (Kish-Gephart et al., 2010; Treviño et al., 2006).

Applied specifically to fraud, Dorminey et al. (2012) traced the evolution from Cressey’s original Fraud Triangle to contemporary elaborations such as Wolfe and Hermanson (2004) fraud diamond, which adds capability to the classical dimensions of pressure, opportunity, and rationalization; Moore et al. (2006) and Murphy and Dacin (2011) further specify the psychological pathways—moral seduction, self-deception, and strategic issue cycling—through which conflicts of interest are transformed into fraudulent action. Together, these three traditions justify the review’s integrative coding framework, which classifies each included study according to whether it addresses a cognitive-heuristic bias, a moral-regulatory mechanism, an organizational-level enabler, or a combination thereof.

Later elaborations extend the model further. The fraud pentagon, developed in professional practice and reviewed by Vousinas (2019), adds arrogance and competence to the elements of the diamond, and Vousinas’s S.C.O.R.E. model (stimulus, capability, opportunity, rationalization and ego) incorporates collusion as a sixth element in the so-called fraud hexagon. The MICE model, in turn, decomposes motivation into money, ideology, coercion and ego (Kranacher et al., 2011). These extensions are relevant here because several of their added elements are psychological in nature: arrogance and ego overlap conceptually with overconfidence and hubris, capability and competence with illusion of control, and collusion with social learning and the normalization of deviance. The review retains the original Fraud Triangle as its primary integrative frame because its three dimensions are the most consistently operationalized across the included studies and because the psychological elements added by later models can be mapped onto these dimensions; the mapped constructs are interpreted in light of the extensions in Section 4.2.

Within white-collar criminology, the main contemporary alternative to the Fraud Triangle is convenience theory, which explains white-collar offending through three dimensions: a financial motive, an organizational opportunity and a personal willingness for deviant behavior (Gottschalk, 2017; Stadler & Gottschalk, 2022). Its behavioral dimension overlaps substantially with the rationalization and moral-disengagement mechanisms examined here. We retain the Fraud Triangle as the primary frame because it is the framework most frequently used by the included studies across accounting, finance and criminology, whereas convenience theory has been tested empirically in comparatively few studies; convenience theory is nonetheless used in the Discussion as a complementary lens for the organizational dimension of offending.

Each tradition also implies a distinct type of evidence and explanatory claim, which the Discussion revisits. The criminological tradition predicts that misconduct varies with perceived sanction certainty and opportunity, so its empirical signature is the association between monitoring, detection and offending (Section 4.3). The neutralization and moral-disengagement tradition predict that misconduct depends on justifications that protect the offender’s moral self-image, so its signature is the presence of rationalization, ethical fading and moral disengagement in offenders’ accounts and in experimental choices (Section 4.2). The organizational-wrongdoing tradition predicts that individual cognition is shaped and aggregated by collective norms and structures, so its signature is the moderation of individual mechanisms by organizational context (Section 4.3 and Section 4.4). The research agenda in Section 4.8 is organized around these three traditions.

### 2.2. Search Strategy

The original database search was conducted on 5 May 2026 in the Web of Science (WoS) Core Collection, selected for its multidisciplinary coverage and curated indexing standards (Birkle et al., 2020). In response to peer review, the search was updated on 1 October 2026 and complemented with a search in Scopus on the same date, given evidence that the two databases differ substantially in their coverage of the social sciences (Mongeon & Paul-Hus, 2016). The following Boolean search string was applied in WoS to titles, abstracts and keywords (field tag TS):

TS = (“white collar crime” OR “white-collar crime” OR “corporate crime” OR “financial crime” OR “occupational crime” OR “organizational crime” OR “corporate fraud” OR “financial misconduct” OR “economic crime”) AND TS = (“cognitive bias*” OR heuristic* OR “bounded rationality” OR overconfidence OR “moral disengagement” OR rationalization OR “ethical fading” OR “motivated reasoning” OR “self-serving bias” OR framing OR “risk perception” OR “decision-making”).

The equivalent Scopus string, applied with the TITLE-ABS-KEY field code, was:

TITLE-ABS-KEY (“white collar crime” OR “white-collar crime” OR “corporate crime” OR “financial crime” OR “occupational crime” OR “organizational crime” OR “organisational crime” OR “corporate fraud” OR “financial misconduct” OR “economic crime”) AND TITLE-ABS-KEY (“cognitive bias” OR heuristic OR “bounded rationality” OR overconfidence OR “moral disengagement” OR rationalization OR rationalisation OR “ethical fading” OR “motivated reasoning” OR “self-serving bias” OR framing OR “risk perception” OR “decision-making”).

**Figure 1:**
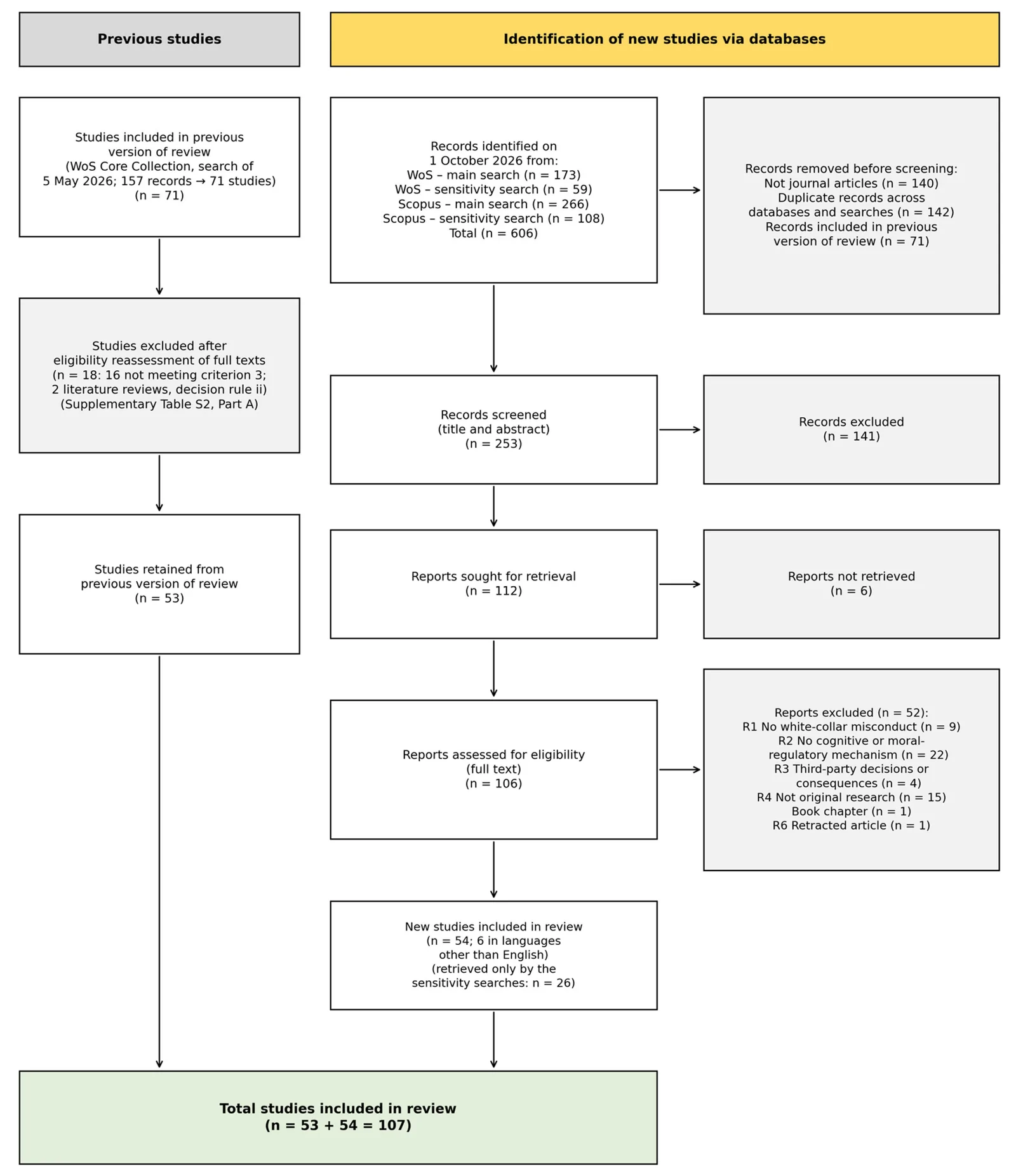
PRISMA 2020 flow diagram for updated systematic reviews: original WoS search, eligibility reassessment of the original studies, updated WoS search, Scopus search and sensitivity search.

British spelling variants (organisational, rationalisation) were added to the Scopus string because WoS applies automatic lemmatization of spelling variants, whereas Scopus does not do so consistently. Document-type restrictions were applied after retrieval in both databases, so that each filtering step could be documented in the PRISMA flow diagram (Figure 1).

The search string was constructed to capture two distinct, theoretically justified categories of terms rather than a single undifferentiated set. The first category (overconfidence, moral disengagement, framing, self-serving bias) corresponds to cognitive biases and heuristics in the strict sense defined by the heuristics-and-biases research program (Tversky & Kahneman, 1974; Kahneman & Tversky, 1979, 1984). The second category (rationalization, ethical fading, risk perception, bounded rationality, decision-making) corresponds to the related psychological and moral-regulatory mechanisms discussed in Section 2.1, which the criminological and behavioral-ethics literatures treat as the proximal pathways linking cognitive distortion to unethical or unlawful conduct (Sykes & Matza, 1957; Tenbrunsel & Messick, 2004; Bazerman & Tenbrunsel, 2011). Both categories were retained within a single search string, and coded separately during data extraction, because restricting the search to strict cognitive-bias terminology alone would have excluded a substantial share of the empirical literature that operationalizes these constructs jointly, thereby reproducing the very fragmentation this review seeks to address. This dual-category logic follows, and extends to a criminological context, the boundary work of prior cognitive-bias reviews in the management literature (Acciarini et al., 2021).

To assess whether the search string omitted psychological constructs that are also associated with white-collar offending—such as dark personality traits, self-deception, cognitive dissonance and dishonesty—a sensitivity search was run in both databases on the same date. It combined the first block of the main string with the terms “dark triad” OR psychopath OR narcissis OR machiavellian OR “self-deception” OR “cognitive dissonance” OR dishonest. Records retrieved only by the sensitivity search were screened with the same eligibility criteria. This search retrieved 67 records that had not been retrieved by the main searches, of which 23 met the inclusion criteria (26 including three non-English records) and were added to the corpus.

### 2.3. Screening and Selection Process

Studies were eligible for inclusion if they met all of the following criteria: (1) reported original empirical research (quantitative, qualitative, or mixed-methods) or a substantive conceptual/theoretical contribution to the cognitive-bias or moral-regulatory literature on white-collar, corporate, or financial misconduct; (2) were published as peer-reviewed journal articles (including articles dually indexed as conference proceedings, but excluding proceedings-only papers, book chapters, editorials, and other non-article document types); (3) directly examined or theorized about a cognitive bias, heuristic, or related psychological/moral-regulatory mechanism (e.g., rationalization, moral disengagement, ethical fading) in relation to white-collar, corporate, financial, occupational, or organizational misconduct; (4) were indexed in the Web of Science Core Collection or Scopus. No restriction was applied to publication year. Studies were excluded if they addressed general ethical decision-making without a criminal or misconduct-related outcome, or if they lacked substantive empirical or conceptual engagement with a specific cognitive bias or psychological mechanism. In the original review, eligibility was restricted to English-language publications. In response to peer review, this restriction was removed: articles in any language indexed in WoS or Scopus were eligible, and full texts in languages not read by the review team were assessed with the assistance of a large language model (Claude, Anthropic), and every decision was verified by one author (J.D.-P.). Because the searches used English terms, non-English articles were retrieved only when their records included English titles, abstracts or keywords. The removal of the language restriction was not part of the registered amendment and is reported as a protocol deviation (Section 2.7).

The study selection process is summarized in Figure 1, which follows the PRISMA 2020 flow diagram for updated systematic reviews. The original WoS search (5 May 2026) was conducted without time restriction to capture the historical development of the field and yielded 157 records. During the identification stage, 15 records were excluded due to document type (10 proceedings papers and five book chapters), resulting in 142 journal articles. During the screening stage, seven articles published beyond the predefined temporal scope were excluded, leaving 135 articles for eligibility assessment. Full-text evaluation resulted in the exclusion of 64 articles that either addressed general ethical decision-making without criminal or misconduct-related outcomes or lacked substantive engagement with a specific psychological mechanism, and the original review retained 71 studies, both empirical and conceptual.

During the preparation of this revision, the eligibility of these 71 studies was reassessed against inclusion criterion 3 by two reviewers (J.D.-P. and C.Q.-B.) on the basis of full texts. This reassessment showed that 16 studies examined the decision-making of actors responding to misconduct—judges, prosecutors, victims or the media—or the consequences of misconduct, rather than a psychological mechanism underlying its commission; these studies had been retained in the original review because the term “decision-making” in the search string retrieved them and the criterion had been applied too broadly. They were therefore excluded, with study-level reasons reported in Supplementary Table S2, leaving 55 studies from the original review. Applying decision rule (ii), described below, to the original corpus led to the exclusion of two further studies that were literature reviews (Schnatterly et al., 2018; Baker & Piquero, 2010), so that 53 studies were retained from the original review.

The updated WoS search, the Scopus search and the sensitivity searches in both databases were conducted on 1 October 2026 and retrieved 173, 266, 59 and 108 records, respectively (606 in total). After restricting the four result sets to English-language journal articles (440 records), 138 cross-database duplicates and the 71 records of the original review were removed, leaving 231 records for title and abstract screening, 67 of which were retrieved only by the sensitivity searches. At the title-abstract stage, 127 records were excluded and 104 were retained for full-text assessment, a record being retained when at least one reviewer rated it as included or unsure. Because the export of the original May 2026 search was not retained, all records not included in the original review were screened anew, including those previously assessed. Of the 104 reports sought, 6 could not be retrieved.

The remaining 98 full texts were assessed for eligibility; 47 were excluded, including one retracted article, with study-level reasons recorded (Supplementary Table S2), and 51 studies were initially included. When decision rule (ii) was refined during the revision (see below), the two parts of a two-part review essay (Pardue et al., 2013a, 2013b) were excluded as literature reviews, and one record was excluded as a chapter published in a book series (Kantšukov & Medvedskaja, 2013), leaving 48 new studies from the English-language records, 23 of which had been retrieved only by the sensitivity searches. When the two reviewers gave different reasons for an exclusion, they both supported, the reason was assigned following the hierarchy R1 > R4 > R3 > R2 for reporting purposes. Together with the six studies identified among the non-English records (see below), the final corpus comprises 107 studies published between 1975 and 2026: 53 retained from the original review and 54 new studies.

Following the removal of the language restriction, the 26 non-English journal articles retrieved by the four searches (22 after deduplication; 9 in Spanish, 4 in French, 3 in Chinese, 2 in Russian and one each in German, Icelandic, Slovene and Ukrainian) were screened with the same criteria and decision rules. Because these records were added late in the revision, title-abstract screening and full-text assessment were carried out with the assistance of a large language model (Claude, Anthropic), which proposed each decision with its justification and page references, and every decision was verified by one author (J.D.-P.); inter-rater agreement is therefore not reported for these records (Section 2.7). Fourteen records were excluded at the title-abstract stage. All eight full texts were retrieved and assessed; two were excluded—a systematic review (Mensch & Rettenberger, 2015) and an article that addressed fraud without examining a psychological mechanism—and six were included: four in French or Spanish conceptual or qualitative contributions on narcissism, psychopathy and organizational biases, one Icelandic survey on personality and tax evasion, and one Spanish-language theoretical framework of organizational corruption (Supplementary Tables S2 and S3).

In the original review, study selection was conducted by four reviewers with complementary disciplinary expertise (J.D.-P., P.S.-G., N.L.-S. and M.T.E.-J.). Eligibility decisions were made collaboratively, and disagreements were resolved through group discussion and consensus. Because decisions were reached jointly rather than independently, inter-rater agreement could not be computed for the original screening (Section 2.7). For the updated and supplementary searches, two reviewers (P.S.-G. and M.T.E.-J.) screened all records independently at the title-abstract and full-text stages, and disagreements were resolved by a third reviewer (J.D.-P.). Agreement was 98.3% at the title-abstract stage (Cohen’s κ = 0.97) and 69.8% at the full-text stage (κ = 0.40) (Cohen, 1960).

Most full-text disagreements concerned two interpretive questions, which were resolved by explicit decision rules applied to all full-text records: (i) studies of dispositional traits (dark triad, corporate psychopathy, narcissism) were included when they linked the trait to white-collar misconduct or to a cognitive or moral-regulatory mechanism, and excluded when they examined the trait without reference to misconduct; and (ii) literature reviews whose primary aim was to summarize existing research—systematic, bibliometric or narrative—were excluded as secondary studies, whereas articles that reviewed the literature in order to develop an original model or framework, and original theoretical essays, were retained as conceptual contributions. The same rules were applied to the studies retained from the original review, which led to the exclusion of the two literature reviews mentioned above (Supplementary Table S2, Part A).

### 2.4. Data Extraction and Coding Procedure

A structured coding framework was developed to systematically extract and classify information from each of the 107 studies included in the final sample. The coding protocol was designed to ensure methodological consistency, transparency, and comparability across disciplines, given the multidisciplinary nature of research on cognitive biases in white-collar crime.

Because the coding records of the original review were not retained, mechanism coding was carried out anew for all 107 included studies using the framework described above, extended with the dispositional-traits category. For each study, a preliminary coding was drafted with the assistance of a large language model (Claude, Anthropic) on the basis of the title and abstract. Two authors (P.S.-G. and M.T.E.-J.) reviewed and, where necessary, corrected every entry, and disagreements were resolved by consensus. Mechanism coding was therefore based mainly on titles and abstracts rather than on full-text extraction (Section 2.7); the six non-English studies were coded from their full texts, and entries for studies whose full texts were read during the quality appraisal were corrected where the full text contradicted the abstract-based coding (for example, the study design, setting or interaction pattern). The coding of each study is reported in Supplementary Table S3.

For each article, detailed information was recorded regarding publication year, country or regional setting, methodological approach, level of analysis, type of misconduct examined, cognitive bias investigated, proximal psychological mechanism identified, and any organizational or situational moderators discussed. Theoretical frameworks applied in each study were also coded, including references to the Fraud Triangle, neutralization theory, moral disengagement theory, rational choice perspectives, and related psychological or criminological models.

To facilitate cross-study synthesis, individual cognitive biases were grouped into broader conceptual categories based on shared psychological functions. Risk perception distortions included overconfidence and illusion of control. Moral self-regulation failures encompassed rationalization, neutralization, moral disengagement, and ethical fading. Motivational distortions captured performance pressure and escalation of commitment, while social learning mechanisms incorporated normalization of deviance and imitation processes. This categorization enabled systematic comparison while preserving theoretical specificity. Following the inclusion of studies on dark personality through the sensitivity searches, a fifth category, dispositional traits, was added to capture stable individual differences such as the dark triad, psychopathy, narcissism, Machiavellianism and low self-control; studies coded in the original review were re-examined against this category.

The coding process was iterative. Initial classifications were reviewed and refined to ensure conceptual coherence and to avoid overlap between categories. Where studies examined multiple psychological mechanisms, all relevant constructs were coded to capture interaction effects.

### 2.5. Quality Appraisal

The methodological quality of the included studies was appraised following the protocol amendment described in Section 2.1. Empirical studies were appraised with the Mixed Methods Appraisal Tool (MMAT, 2018 version), which provides design-specific criteria for qualitative, quantitative randomized, quantitative non-randomized, quantitative descriptive and mixed-methods studies (Hong et al., 2018). Conceptual and theoretical contributions were appraised with the JBI Critical Appraisal Checklist for Text and Opinion (McArthur et al., 2015). In line with MMAT guidance, no overall numerical score was computed; the criteria met by each study are reported in Supplementary Table S1.

One reviewer (C.Q.-B.) appraised the 53 studies retained from the original review, recording a justification for each criterion rated “No” or “Can’t tell”. A second reviewer (N.L.-S.) verified every rating against the full text, and disagreements were resolved by consensus; the changes agreed by consensus are documented in Supplementary Table S1. Because the verifying reviewer had access to the first reviewer’s ratings, the two appraisals were not independent, and inter-rater agreement is therefore not reported for this stage (Section 2.7). Appraisal results were not used to exclude studies. They were used descriptively and in a sensitivity check that examined whether the main thematic conclusions held when studies meeting fewer than three of the five MMAT criteria were excluded (Section 3.1).

For the 54 studies added in this revision, a large language model (Claude, Anthropic) was used as a structured extraction aid. Working from the full texts, it proposed the design category and a rating for each criterion, together with the supporting passage and its page reference, applying the decision rules agreed for the original corpus. The model did not make final decisions: one author (J.D.-P.) checked each proposal against the full text and assigned the final rating. During this step, three studies were reclassified (two from conceptual to qualitative and one from qualitative to conceptual). Three added studies whose full texts could not be accessed were not appraised (Section 2.7).

### 2.6. Analytical Approach

The first stage of analysis involved descriptive bibliometric assessment of the field. Publication trends were examined across the period from 1975 to 1 October 2026 in order to identify developmental phases and growth patterns. Geographic distribution of research activity was analyzed to assess regional concentration and international collaboration. This descriptive phase provided a structural overview of how cognitive bias research in white-collar crime has evolved over time and across academic domains.

The second stage consisted of a thematic synthesis of the included studies. The primary objective was to identify the most frequently examined cognitive biases and to determine how these biases were operationalized across empirical contexts. Particular attention was given to distinguishing between biases affecting risk perception, moral cognition, motivational processing, and social norm internalization. Thematic analysis further explored how cognitive distortions interacted with organizational and situational factors, including governance quality, monitoring intensity, compensation structures, regulatory environments, and cultural norms. Studies were also examined to determine whether they addressed misconduct commission, detection probability, escalation, or concealment processes.

The final analytical stage involved integrating empirical findings into a unified conceptual framework. Cognitive distortions were mapped onto proximal psychological processes such as risk underestimation, guilt reduction, ethical detachment, and motivational pressure internalization. These processes were then aligned with the dimensions of the Fraud Triangle—pressure, opportunity, and rationalization—to clarify how biases contribute to criminal outcomes.

Because the corpus combines quantitative, qualitative and conceptual studies, evidence was integrated using a convergent segregated approach adapted from JBI guidance for mixed-methods systematic reviews (Stern et al., 2020). Findings were first extracted separately by evidence type: quantitative studies were coded for the direction and significance of the reported association between a mechanism and a misconduct outcome; qualitative studies for the mechanism described in offenders’ or professionals’ accounts; and conceptual studies for the pathway they proposed. These findings were then aligned at the level of constructs in a construct-by-evidence-type matrix. A mechanism was classified as a dominant category when it was supported by studies of at least two evidence types, and an interaction pattern (amplification, attenuation, reinforcement or mediation) was reported only when at least one empirical study supported it. This procedure does not resolve the general lack of consensus on how heterogeneous evidence should be combined, which is acknowledged in Section 2.7, but it makes the integration criteria explicit and replicable.

Bibliometric analyses were conducted on the final corpus of 107 studies with the Bibliometrix package (R) through its Biblioshiny interface (version 4.3.0) (Aria & Cuccurullo, 2017). Bibliographic records were drawn from the WoS and Scopus exports and combined into a single file in WoS format; for studies indexed in both databases, the record with the more complete metadata was used. Country affiliations were taken from the author affiliation fields; for 21 studies whose WoS records listed institutions without countries, the country of each institution was assigned manually, and Hong Kong and Macao were counted as China. The country collaboration map shows each country’s number of studies and the co-authorship links between countries. The thematic network was built as a co-occurrence network of author keywords (or, where these were missing, database-assigned keywords), after harmonizing spelling variants and synonyms (e.g., white collar crime/white-collar crime; rationalisation/rationalization; corporate psychopaths/corporate psychopathy). The network was restricted to the 15 most frequent keywords, corresponding to terms occurring at least five times, with association normalization, Louvain clustering and removal of isolated nodes.

Because this review reports a thematic and bibliometric synthesis rather than a quantitative pooling of effect sizes, several PRISMA 2020 items concerned with meta-analysis do not apply and are addressed here for completeness. No effect measures were defined or pooled (cf. PRISMA item 12); no meta-analytic model, heterogeneity statistic, or sensitivity analysis was used to combine results across studies (items 13e–13f); no study-level or synthesis-level statistical results, forest plots, or precision estimates are reported (items 19, 20b–20d); no formal assessment of reporting bias (e.g., funnel plot or Egger’s test) or of certainty/confidence in a pooled body of evidence (e.g., GRADE) was performed, since these require a quantitative synthesis that this design does not produce (items 14, 15, 21, 22). The eligible studies identified under Section 2.3 were grouped for synthesis according to the cognitive-bias and moral-regulatory categories defined in Section 2.4, consistent with the eligibility criteria in Section 2.3 (item 13a). Thematic coding and categorization (Section 2.4) were performed by the review team using a shared spreadsheet; bibliometric mapping specifically used the Bibliometrix package in R (Aria & Cuccurullo, 2017), as noted above (item 13d).

### 2.7. Methodological Limitations

The original review did not include a formal quality appraisal; this was added after peer review (Section 2.5). Because the appraisal was conducted after the synthesis categories had been defined, it could inform the interpretation of the findings but not the initial coding.

Several methodological limitations warrant consideration. First, although the search now covers both the WoS Core Collection and Scopus, relevant studies published in regional or non-indexed outlets, in books or in the grey literature may not have been captured. No systematic search of grey literature or doctoral dissertations was undertaken.

Second, publication bias may influence the available evidence, as studies reporting significant associations between cognitive biases and misconduct are more likely to be published. Funnel-plot-based tests do not apply to a thematic synthesis, but the implications for the present conclusions are substantive: the predominance of moral self-regulation failures and dispositional traits should be read as their predominance in the published literature rather than as a definitive ranking of their causal importance, the strength of the amplification and attenuation patterns may be overstated, and mechanisms with mixed or null evidence may be underrepresented.

Third, operationalization of cognitive biases varies considerably across disciplines. In accounting and finance research, biases are often inferred from behavioral proxies, such as executive decision patterns or observable traits, rather than measured directly using validated psychological instruments. This heterogeneity complicates cross-study comparability and constrains precise estimation of psychological mechanisms. This heterogeneity also raises questions of cross-cultural measurement equivalence, discussed in Section 4.7.

Fourth, as with all systematic reviews, conclusions are limited by the methodological rigor of the included studies. Variation in research design, sample characteristics, and measurement strategies may affect the strength and consistency of reported relationships. A related and more fundamental limitation is the methodological heterogeneity of the evidence base. The included studies combine experimental, survey-based, archival, qualitative and conceptual designs, analysed with markedly different techniques (e.g., regression and panel models, structural equation modelling, content and narrative analysis) that rely on different operationalizations of both the psychological mechanisms and the misconduct outcomes. To date, the evidence-synthesis literature has not reached consensus on how findings produced by such divergent approaches should be combined, and no available integration procedure fully overcomes this problem. The convergent segregated approach described in Section 2.6 makes the integration criteria explicit and replicable, but it does not remove this limitation. Consequently, the findings cannot be aggregated statistically or compared in terms of effect magnitude, and the dominant categories and interaction patterns reported in Section 3 should be read as convergent patterns across heterogeneous evidence rather than as estimates of the relative strength of each mechanism. As the empirical base matures, more standardized measures of cognitive biases in misconduct contexts would make meta-analytic designs restricted to methodologically comparable studies feasible.

Fifth, although no language restriction was applied in the final eligibility criteria, the searches were conducted with English terms in two international databases. Non-English articles were therefore retrieved only when indexed in WoS or Scopus with English metadata, and Spanish- and Portuguese-language scholarship on corruption, corporate fraud and compliance in Latin America published in regional journals not covered by these databases is likely under-represented. Given that the author team is based in Chile and Ecuador, this is a consequential gap, and it reinforces the under-representation of Global South contexts documented in Section 3.2. Future replications should add searches in Spanish and Portuguese and regional databases such as SciELO and Redalyc. In addition, full texts in some languages were assessed with machine translation, which may have reduced the precision of the assessment of these studies.

Sixth, the original stages of the review were registered retrospectively, after screening and data extraction had been completed, rather than prospectively in a registry such as PROSPERO or OSF before the search. Retrospective registration offers weaker protection against outcome-driven decisions about eligibility and synthesis, and readers should weigh the original stages accordingly. The extensions introduced after peer review were registered before their execution (Section 2.1). In addition, the reassessment of the eligibility of the originally included studies (Section 2.3) was decided after these studies had been examined and was not part of the registered amendment; it is therefore reported as a deviation from the registered protocol. Because it was applied uniformly to all originally included studies using the unchanged inclusion criterion 3, and every exclusion is documented with reasons, we consider the risk of selective exclusion to be low, but readers should take it into account.

Seventh, inter-rater reliability could not be quantified for the original screening and coding, because decisions were reached jointly by consensus rather than independently, nor for the quality appraisal, which was conducted by one reviewer and verified by a second rather than performed independently. Agreement statistics are therefore available only for the screening of the updated searches. The quality appraisal procedure also departs from the registered amendment, which specified independent double appraisal; this is reported as a deviation from the protocol.

Eighth, the corpus of 107 studies is moderate in size. This reflects the deliberately narrow intersection targeted by the review—cognitive or moral-regulatory mechanisms and white-collar misconduct—rather than the broader fields of cognitive bias or white-collar crime, each of which comprises thousands of publications. The bibliometric component should therefore be read as a descriptive map of a specialized subfield; network statistics based on a corpus of this size are sensitive to individual studies and to threshold choices.

Ninth, because the coding records of the original review were not retained, the mechanism coding of the 53 studies retained from that review was based on titles and abstracts, with a preliminary draft prepared with the assistance of a large language model and reviewed by two authors, rather than on systematic full-text extraction. For the added studies, whose full texts were read during screening and quality appraisal, entries were corrected where the full text contradicted the abstract-based coding, and the six non-English studies were coded from their full texts. Mechanisms discussed only in the body of an article may nonetheless be under-represented, so the category frequencies reported in Section 3.4 should be read as approximate. Two features of the results limit the consequences for the main conclusion: the two leading categories (33 studies each) are separated by a wide margin from the next (16 studies), so a small number of misclassified studies would not change their ranking, and the ranking was unchanged when the lower-quality studies were excluded (Section 3.1). Eligibility decisions at the full-text stage and the quality appraisal were based on the full texts.

Tenth, the quality appraisal of the studies added in this revision, and the screening of the non-English records, were not performed by two independent human reviewers, as specified in the registered amendment. A large language model was used as a structured extraction aid that proposed each rating or decision with the supporting passage and page reference, under the decision rules agreed for the original corpus, and the final decision was made by one author (J.D.-P.) after checking each proposal against the full text. Because only one author verified these proposals, inter-rater agreement cannot be reported for these steps, and this is reported as a deviation from the protocol. Three added studies whose full texts could not be accessed were not appraised.

For the original review, an itemized record of the 64 articles excluded at the full-text eligibility stage was not preserved. This gap was addressed in the update: all records retrieved by the WoS search that had not been included in the original review were screened anew with the same criteria, so a documented reason for exclusion is now available for every retrieved record that is not part of the final corpus (screening records available from the authors; full-text exclusions in Supplementary Table S2, Part B).

Despite these limitations, the structured, transparent, and multidisciplinary approach adopted in this review enhances methodological reliability and contributes to cumulative knowledge development within criminal psychology.

## 3. Results

### 3.1. Descriptive Overview of the Evidence Base

Study-level appraisal results are reported in Supplementary Table S1, together with each study’s design category, level of analysis, setting and theoretical framework. Of the 42 empirical studies retained from the original review, 9 (all qualitative) met all five MMAT criteria, 11 met four and 22 met three. Of the 28 empirical studies added in this revision, 3 met all five criteria, 7 met four, 4 met three, 7 met two, 5 met one and 2 met none. Across both groups, the criteria most frequently rated as not met or unclear were exposure stability in archival studies (criterion 3.5), blinding and adherence in survey experiments (criteria 2.4 and 2.5), the representativeness of convenience, student and online samples (criterion 3.1) and the appropriateness of bias measures, often based on indirect proxies such as auditor change for rationalization (criterion 3.2). Of the 34 conceptual studies, 16 met all six JBI criteria, 16 met five and 2 met four, the unmet criteria mostly concerning the defense of incongruent evidence (Q6). Three added studies could not be appraised. In the sensitivity check, the 14 empirical studies meeting fewer than three MMAT criteria—all of them added in this revision—were excluded. Among the remaining 93 studies, moral self-regulation failures (31 studies, 33.3%) and dispositional traits (25, 26.9%) remained the two most frequent primary mechanisms, followed by social learning mechanisms (14), motivational distortions (12) and risk-perception distortions (11), so the main thematic conclusion did not depend on the lower-quality studies.

**Figure 2:**
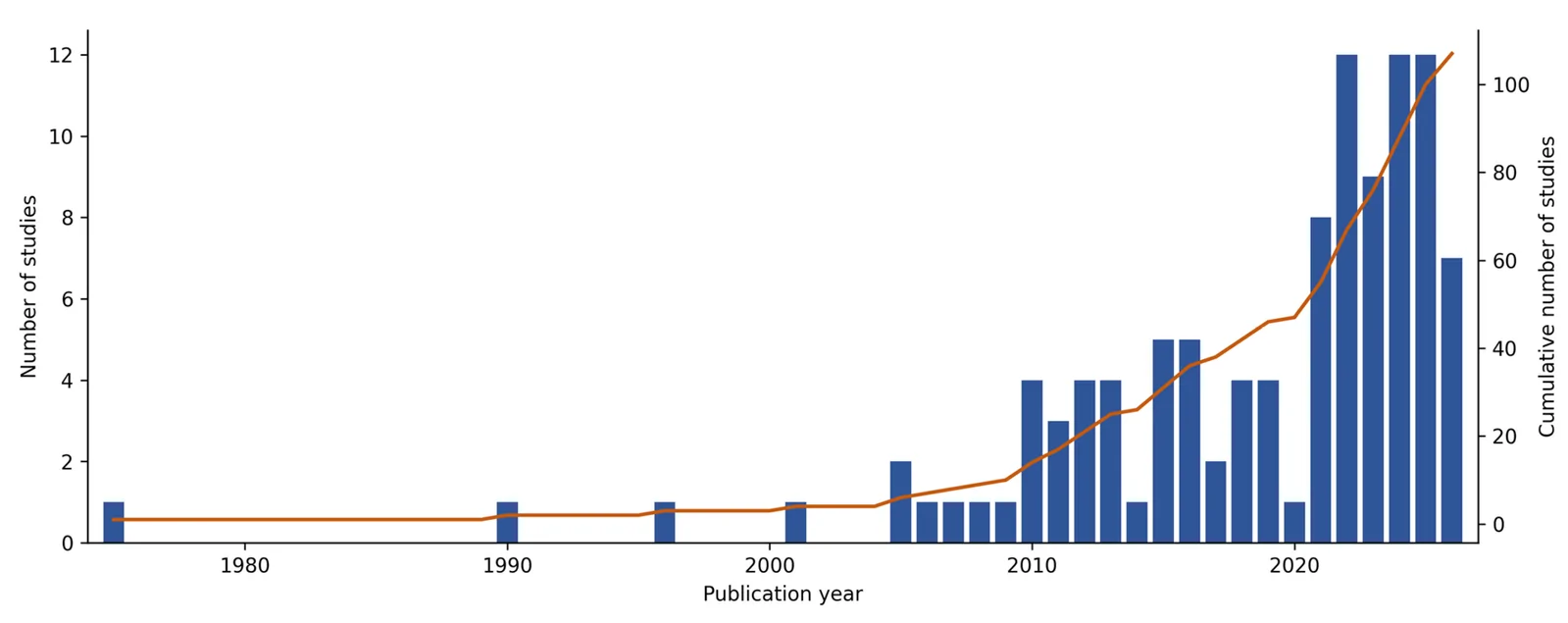
Annual and cumulative number of included studies (1975–2026; n = 107). Blue bars represent the annual number of included studies, while the red line indicates the cumulative number of studies over the period. Data for 2026 cover 1 January–1 October only.

Figure 2 shows the temporal evolution of the 107 included studies, published between 1975 and 1 October 2026. Three phases can be distinguished. In the early phase (1975–2008), contributions were sporadic (9 studies, 8.4% of the corpus) and largely conceptual or exploratory, reflecting foundational attempts to apply rational choice theory, the Fraud Triangle and neutralization theory to white-collar offending.

In the second phase (2009–2018), research activity increased steadily (33 studies, 30.8%). This growth coincides with the aftermath of the global financial crisis, during which public and academic attention to corporate misconduct intensified, and with the emergence of research on corporate psychopathy and narcissistic leadership.

In the third phase (2019–2026), publication frequency accelerated markedly: 65 studies (60.7%) appeared in this period, with peaks of 12 studies in 2022, 2024 and 2025. This surge suggests the consolidation of the psychology of white-collar crime as a distinct line of research, in which behavioral economics, corporate governance and criminal psychology increasingly intersect.

From a criminal psychology perspective, this temporal growth indicates a paradigmatic shift: misconduct is increasingly conceptualized not solely as a function of external pressures, but as the outcome of predictable cognitive vulnerabilities operating within organizational contexts.

### 3.2. Geographic Distribution and International Collaboration

**Figure 3:**
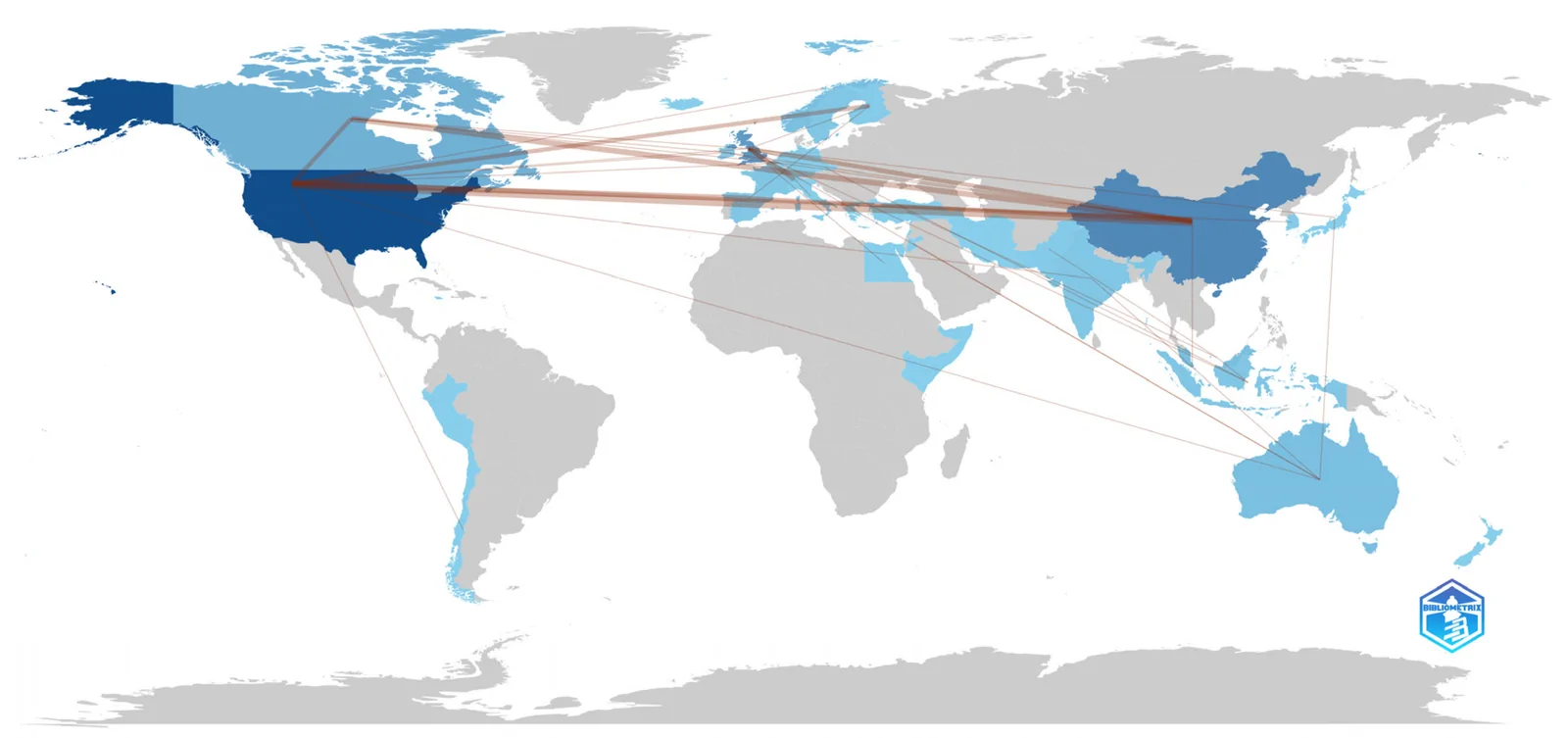
Country collaboration map of the included studies (n = 107). Colour intensity reflects the number of studies per country; lines indicate co-authorship links.

Figure 3 maps the countries of the authors’ affiliations and their collaboration links. The 107 studies involve authors from 38 countries. The United States is the main research hub (37 studies, 34.6%), followed by China (19), the United Kingdom (15), Canada (9), and Australia, Malaysia and Norway (5 each).

Twenty-eight studies (26.2%) involved authors from more than one country. The strongest collaboration ties link the United States with China (6 co-authored studies) and France (3), and China with Canada (3). The United Kingdom acts as a second bridge, connecting European, Asian and Australian institutions, and a smaller regional cluster links Malaysia, Indonesia, Pakistan and Turkey. Twelve countries, including Germany, Spain and Sweden, contributed only single-country studies. Latin America is represented by two studies (Chile and Peru), Africa by three (Egypt, Kenya and Somalia), and the Middle East by four (Iran, Jordan, Lebanon and Turkey).

This geographic clustering has important theoretical implications. Cognitive bias research in white-collar crime is disproportionately shaped by institutional contexts characterized by developed regulatory systems, capital market transparency, and formalized governance structures. Consequently, the psychological mechanisms identified in the literature may reflect context-specific dynamics.

For criminal psychology, this raises critical questions regarding cross-cultural generalizability. Cognitive distortions such as overconfidence, rationalization, and moral disengagement may manifest differently under varying enforcement regimes, collectivist versus individualist cultures, and divergent corruption norms. The limited representation of Global South contexts represents a significant empirical gap. Thus, Figure 3 not only illustrates collaboration patterns but also highlights structural imbalances in the production of psychological knowledge about corporate misconduct.

### 3.3. Intellectual and Thematic Clustering

**Figure 4:**
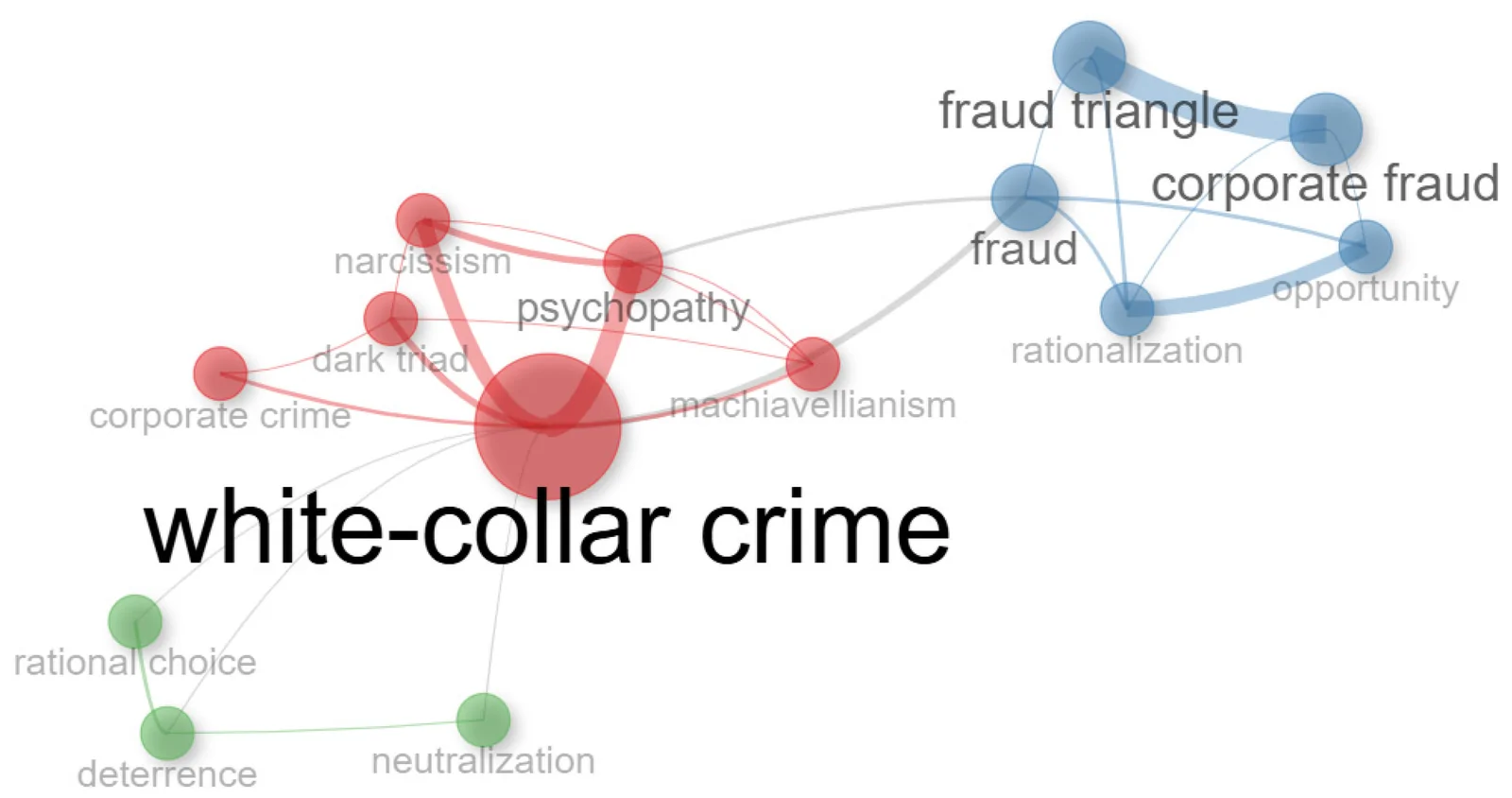
Keyword co-occurrence network of the included studies (author keywords occurring at least five times; Louvain clustering). Node colours represent three thematic clusters: red indicates white-collar crime and dark personality traits (narcissism, psychopathy, Machiavellianism, and the dark triad); blue represents corporate fraud and the fraud triangle (opportunity and rationalization); and green denotes rational choice, deterrence, and neutralization perspectives. Node size reflects keyword frequency, while links indicate keyword co-occurrence, with thicker links representing stronger relationships.

Figure 4 presents the keyword co-occurrence network, in which 14 frequent keywords formed three clusters (one further keyword, business ethics, was not retained in the network). The first cluster (blue) is organized around the Fraud Triangle and corporate fraud and includes rationalization and opportunity; it is rooted mainly in accounting, finance and fraud-examination research and frequently relies on archival data and the Fraud Triangle and its extensions.

The second cluster (red) is organized around white-collar and corporate crime and dark personality traits (psychopathy, narcissism, Machiavellianism and the dark triad). It is rooted mainly in criminology and personality psychology and has grown substantially with the studies added in this revision. The third cluster (green) links deterrence, rational choice and neutralization, reflecting the criminological tradition of offender decision-making.

The clusters are connected mainly through the general term “white-collar crime”, whereas direct ties between their specific constructs are sparse: Fraud Triangle constructs rarely co-occur with personality constructs, and neither co-occurs frequently with deterrence and neutralization. Notably, overconfidence and moral disengagement, which are central to the thematic synthesis, fall below the five-occurrence threshold, indicating that these mechanisms are often studied under broader labels such as fraud, rationalization or personality.

For criminal psychology, this fragmentation is particularly salient. The Fraud Triangle cluster emphasizes situational pressures and justifications, the personality cluster emphasizes stable dispositions, and the deterrence cluster emphasizes the perceived costs and benefits of offending. Yet these mechanisms may operate simultaneously within the same decision-making context.

The existence of these clusters strengthens the rationale for the integrative framework proposed in this review. By bridging moral disengagement and risk-based distortions within a unified cognitive–organizational pathway, this study advances a more comprehensive psychological explanation of white-collar misconduct.

### 3.4. Core Cognitive Biases in White-Collar Crime

Across the 107 included studies, the primary mechanism examined was a moral self-regulation failure in 33 studies (30.8%), a dispositional trait in 33 (30.8%), a motivational distortion in 16 (15.0%), a social learning mechanism in 14 (13.1%) and a risk-perception distortion in 11 (10.3%) (Supplementary Table S3). The composition of the corpus changed markedly with the updated searches: dispositional traits are the primary mechanism in 26 of the 54 added studies but in only 7 of the 53 studies retained from the original review, whereas risk-perception distortions such as overconfidence are concentrated in the original, mostly archival, corporate-fraud studies. This pattern held in the sensitivity analysis excluding lower-quality studies (Section 3.1). Table 1 synthesizes the main mechanisms and their representative evidence.

Overconfidence appears most frequently in corporate and financial contexts, particularly in studies examining executive decision-making, earnings management, and fraud commission. Its psychological function is primarily epistemic: it distorts risk perception and inflates perceived control. From a criminal psychology perspective, overconfidence reduces perceived sanction certainty and amplifies opportunity recognition, thereby increasing the likelihood of misconduct in ambiguous regulatory environments.

Rationalization mechanisms, grounded in neutralization theory, constitute the second dominant category. Unlike overconfidence, which affects cognitive risk assessment, rationalization operates on moral self-regulation. It reduces anticipatory guilt and preserves moral identity, allowing actors to reinterpret unethical conduct as acceptable, necessary, or victimless. This aligns closely with psychological accounts of cognitive dissonance reduction and ethical fading.

Moral disengagement overlaps with rationalization but reflects a broader detachment from ethical standards, often involving diffusion of responsibility or dehumanization of stakeholders. Its prominence in greenwashing and environmental misconduct research suggests that ethical detachment may be particularly salient when harm is indirect or abstract.

Importantly, Table 1 also reveals multi-level variation. Some biases operate primarily at the individual executive level (e.g., overconfidence), whereas others emerge in group or cultural contexts (e.g., social learning). This supports a layered understanding of white-collar crime as the product of interacting cognitive and social processes rather than isolated personality traits.

Consistent with the dual-construct scope established in Section 2 and the theoretical distinction developed in Section 2.1, Table 1 separates two analytically distinct categories rather than treating all seven rows as cognitive biases in the strict sense. Overconfidence Bias is a cognitive-heuristic bias proper. Rationalization Mechanisms, Moral Disengagement, and Ethical Sensitivity Differences are moral-regulatory mechanisms that condition how a cognitive distortion, once activated, is acted upon. Performance Pressure Bias, Escalation of Commitment, and Social Learning Bias are organizational and motivational enablers rather than cognitive biases per se; they are retained in the synthesis because the included empirical studies analyze them jointly with cognitive-heuristic and moral-regulatory constructs, and separating them would have fragmented findings that the primary studies themselves report as interdependent. The right-hand column of Table 1 is therefore best read as identifying the psychological function each construct performs within the broader cognitive–organizational pathway to misconduct, not as a claim that each entry is a cognitive bias in the technical sense.

Table 2 demonstrates that cognitive biases rarely operate in isolation; rather, they are systematically shaped by organizational governance structures and situational pressures. The evidence suggests two broad interaction patterns: amplification effects and attenuation effects.

Amplification occurs when structural weaknesses, such as poor board independence, weak internal controls, or permissive organizational culture, intensify the behavioral consequences of cognitive distortions. For example, overconfidence significantly increases fraud likelihood when governance oversight is limited. Similarly, rationalization mechanisms become more potent in environments characterized by group normalization of deviance.

Attenuation, by contrast, is observed when monitoring mechanisms reduce the behavioral expression of biases. Strong analyst coverage, short-selling activity, and audit quality appear to weaken the impact of overconfidence by increasing perceived detection probability.

From a criminal psychology standpoint, this interactional evidence is critical. It demonstrates that psychological vulnerabilities require enabling contexts. Cognitive distortions become criminogenic when embedded within permissive or weakly regulated organizational systems. This finding bridges individual-level cognitive theory with macro-level criminological structures.

Table 3 integrates the empirical findings into a multistage explanatory pathway linking cognitive distortions to specific criminal outcomes. The analysis reveals that biases operate through proximal psychological processes, including: Risk underestimation (overconfidence), Guilt reduction (rationalization), Ethical detachment (moral disengagement), and Motivational pressure internalization (underperformance bias).

These proximal processes activate one or more components of the Fraud Triangle, particularly opportunity and rationalization. Overconfidence primarily activates the opportunity dimension by distorting risk perception, whereas rationalization and moral disengagement directly reinforce the rationalization dimension.

Notably, several studies distinguish between fraud commission and fraud detection. Overconfidence may increase commission by underestimating risk, while weak monitoring reduces detection probability independently of initial bias activation. This differentiation is theoretically significant, as it clarifies whether biases influence criminal intent, execution, or concealment.

From a criminal psychology perspective, Table 3 supports a cognitive-process model of white-collar crime. Misconduct emerges not from static criminal disposition but from predictable distortions in risk evaluation, moral self-regulation, and motivational processing.

Table 4 translates the identified cognitive mechanisms into actionable prevention strategies across multiple levels of intervention. At the governance level, separating CEO and chair roles and strengthening board independence are proposed to counteract overconfidence-driven dominance. Institutional-level transparency mechanisms (e.g., analyst monitoring) function as cognitive correctives by increasing perceived detection probability.

At the organizational level, ethics programs targeting neutralization techniques directly address rationalization processes. This represents a psychologically informed compliance strategy that moves beyond rule-based deterrence.

At the individual level, strengthening accountability frameworks and whistleblowing systems mitigates moral disengagement by reintroducing personal responsibility into decision-making contexts.

Importantly, the table reveals that effective prevention must be bias-specific rather than generic. Overconfidence requires structural oversight; rationalization requires moral reframing; social learning requires cultural intervention.

For criminal psychology, this highlights a shift from reactive punishment to preventive cognitive design. Understanding the mechanisms by which biases operate allows institutions to construct environments that reduce the likelihood that cognitive vulnerabilities will translate into misconduct.

## 4. Discussion

Before discussing the findings in detail, we summarize the answers to the four review questions stated in Section 1.2. Regarding RQ1, moral self-regulation failures (33 studies), mainly rationalization, neutralization and moral disengagement, and dispositional traits (33 studies), mainly psychopathy, narcissism and the dark triad, are the most frequently examined mechanisms, followed by motivational distortions (16), social learning mechanisms (14) and risk-perception distortions such as overconfidence (11) (Section 3.4; Supplementary Table S3). Regarding RQ2, these mechanisms are amplified by performance pressure, unmet aspirations, weak governance and collective norms that legitimize deviance, and attenuated by external monitoring, board independence and credible detection (Table 2). Regarding RQ3, the evidence points to two pathways that map onto the Fraud Triangle and its extensions: a risk-based pathway, in which overconfidence and illusion of control lead offenders to underestimate detection and overestimate their control over opportunities, and a moral-regulatory pathway, in which rationalization and moral disengagement reduce the anticipated guilt of misconduct (Section 4.2). Regarding RQ4, the findings support prevention strategies that target specific mechanisms rather than generic compliance (Section 4.5), and they identify the knowledge gaps summarized in Table 5 (Section 4.8).

These results support the working proposition in its general form: cognitive biases and moral-regulatory mechanisms operate as proximal pathways between pressure, opportunity and rationalization on the one hand and misconduct on the other, and their expression depends on organizational context. The proposition must nonetheless be qualified in three respects. First, the evidence is largely correlational, and many studies infer biases from archival proxies rather than measuring them directly. Second, the aggregation of individual mechanisms into organizational patterns has been theorized more often than tested. Third, it remains unresolved whether these mechanisms constitute an explanatory layer distinct from stable dispositional traits (Section 4.6).

### 4.1. A Cognitive Turn in White-Collar Crime Research

The findings of this systematic review indicate that white-collar crime research is undergoing a significant psychological reorientation. Since Sutherland’s (1949) seminal formulation of white-collar crime as occupational deviance committed by individuals of high social status, explanations have largely emphasized structural opportunity, differential association, and organizational context. Subsequent criminological models, including rational choice theory (R. Clarke & Cornish, 1985), have assumed relatively deliberate and calculative decision-making processes.

However, the synthesis presented here suggests that misconduct frequently arises from systematic distortions in perception, judgment, and moral reasoning. We use the term cognitive in a broad sense. It covers the heuristic and risk-perception biases studied in behavioral decision theory, the processes of moral cognition through which offenders construe, justify and disengage from the ethical content of their decisions, and the stable dispositions that shape both. This broad reading reflects the composition of the evidence: moral self-regulation failures and dispositional traits are each the primary mechanism in 30.8% of the included studies, whereas classic risk-perception biases such as overconfidence account for 10.3% (Section 3.4). The behavioral-decision strand nonetheless remains foundational, particularly the work of Kahneman and Tversky (1979), who demonstrated that human decision-making under uncertainty is shaped by predictable cognitive biases rather than fully rational calculations.

Within corporate and financial contexts, the mechanisms examined most often are moral self-regulation failures and stable dispositional traits. Rationalization reframes misconduct as justified or necessary (Sykes & Matza, 1957), and moral disengagement weakens internal self-sanctions (Bandura, 1999); traits such as psychopathy, narcissism and Machiavellianism appear to increase susceptibility to these processes (Blickle et al., 2006; Ragatz et al., 2012). Risk-perception distortions such as overconfidence, although examined in fewer studies, distort risk estimation and inflate perceived control, particularly in archival research on executive fraud. Together, these mechanisms challenge the assumption that white-collar offenders operate solely as rational utility maximizers and suggest that misconduct often emerges from gradual cognitive shifts embedded in routine decision-making, shaped in part by stable individual differences.

### 4.2. Integrating Cognitive Biases with the Fraud Triangle

A central theoretical contribution of this review lies in clarifying how cognitive biases interact with Cressey’s (1953) Fraud Triangle. The evidence indicates that cognitive distortions map systematically onto the three core dimensions of pressure, opportunity, and rationalization.

Although examined in fewer studies than moral and dispositional mechanisms (Section 3.4), overconfidence primarily activates the opportunity dimension by reducing perceived sanction certainty and inflating executives’ beliefs in their ability to manage legal or reputational risks. This finding extends rational choice assumptions by demonstrating that perceived risk is psychologically distorted rather than objectively calculated (Kahneman & Tversky, 1979).

Rationalization and moral disengagement directly reinforce the rationalization dimension. Neutralization techniques (Sykes & Matza, 1957) and moral disengagement processes (Bandura, 1999) reduce guilt and preserve moral identity, thereby lowering internal barriers to misconduct. Performance pressure and escalation biases correspond to the pressure dimension, transforming financial strain into perceived necessity.

Dispositional traits, the second most frequent category in the expanded corpus, cut across the three dimensions. Entitlement and ego shape how pressure is experienced, manipulativeness and boldness facilitate the recognition and exploitation of opportunity, and callousness and lack of remorse make misconduct easier to rationalize (Lingnau et al., 2017; Ragatz et al., 2012). This reading is consistent with the ego and capability elements of later fraud models discussed below.

These findings suggest that the Fraud Triangle should be understood not merely as a structural model but as a psychologically mediated process. Structural opportunity and economic strain create conditions for misconduct, but cognitive distortions shape how those conditions are interpreted and acted upon.

This reading is consistent with later extensions of the Fraud Triangle. The capability element of the fraud diamond (Wolfe & Hermanson, 2004) and the competence and arrogance elements of the fraud pentagon correspond closely to overconfidence, hubris and illusion of control as coded in this review. The ego element of the S.C.O.R.E. model (Vousinas, 2019) and of the MICE model (Kranacher et al., 2011) captures the self-enhancing motives that underlie both overconfidence and entitlement-based rationalization, while the collusion element of the fraud hexagon corresponds to the social learning and group normalization mechanisms identified in Table 1, Table 2 and Table 3. In this sense, the extensions can be read as partial psychologizations of the triangle, which the present synthesis makes explicit by locating each added element in a specific cognitive or moral-regulatory process.

### 4.3. Contextual Amplification and Organizational Moderation

The review further demonstrates that cognitive biases do not operate in isolation. Their criminogenic potential depends on organizational and institutional context. Weak governance structures, concentrated executive power, and permissive cultures amplify the behavioral consequences of rationalization, overconfidence and dark personality traits. Conversely, strong monitoring mechanisms attenuate their effects by increasing perceived detection probability. In the studies on dark personality, organizational context appears mainly as an enabling condition: governance lapses and weak accountability allow leaders with psychopathic or narcissistic traits to act on them (Murthy & Gopalkrishnan, 2023), whereas external accountability mechanisms are proposed to constrain them (Latif et al., 2026).

This interaction supports a multilevel understanding of white-collar crime consistent with situational crime prevention theory (R. V. Clarke, 1997). Psychological vulnerabilities require enabling contexts to translate into misconduct. Cognitive distortions become criminogenic when embedded within organizational systems that fail to provide corrective feedback or ethical constraints.

For criminal psychology, this reinforces the importance of examining how individual-level cognition interacts with structural moderators. Misconduct emerges not from isolated cognitive bias alone, but from the intersection of biased perception and institutional permissiveness.

The multilevel integration can be specified further. At the individual level, biases such as overconfidence distort risk perception and self-assessment. At the group level, these distortions are aggregated through shared justifications, collective rationalization and the socialization of newcomers into deviant routines (Ashforth & Anand, 2003). At the organizational level, they may crystallize into an organizational cognitive climate in which misconduct is no longer perceived as such, a notion closely related to the established construct of ethical work climate (Victor & Cullen, 1988), a condition captured by the distinction between corrupt organizations and organizations of corrupt individuals (Pinto et al., 2008) and by the notion of normal organizational wrongdoing (Palmer, 2012).

Multilevel theory suggests two ways in which such aggregation may occur (Kozlowski & Klein, 2000). Through composition, similar biases among members—for example, a top management team selected for confidence—produce a shared distortion. Through compilation, distinct individual mechanisms combine into an emergent property, as when an overconfident executive’s decisions are rationalized by subordinates who morally disengage from their consequences. Whether individual biases aggregate into an organizational cognitive climate through either route remains an open empirical question, because few of the included studies measured cognitive constructs at more than one level simultaneously.

### 4.4. Bridging Moral and Risk-Based Distortions

The keyword network reveals a fragmentation into three streams: a Fraud Triangle and corporate-fraud stream rooted in accounting and finance, a dark-personality stream rooted in criminology and personality psychology, and a deterrence and neutralization stream. Moral distortions such as rationalization and neutralization appear in the first and third streams, dispositional traits in the second, and risk-perception distortions such as overconfidence mainly in archival corporate-governance research within the first. Although all three examine psychological sources of misconduct, theoretical integration among them remains limited.

Yet empirical evidence suggests that moral and epistemic distortions often operate simultaneously. An executive may underestimate legal risk due to overconfidence while simultaneously neutralizing ethical concerns through rationalization. Integrating these processes provides a more complete psychological account of misconduct.

From a theoretical standpoint, combining risk perception theory (Kahneman & Tversky, 1979) with moral disengagement theory (Bandura, 1999) advances a unified cognitive–organizational model of white-collar crime. This integration represents a key contribution to criminal psychology by bridging previously siloed research traditions. The growing literature on dark personality adds a third component to this model: stable traits that may predispose individuals both to distorted risk appraisal and to readier moral disengagement (Section 4.6).

Several constructs that are often discussed separately in the literature fall within this bridge between moral and risk-based distortions. Self-deception is at the core of ethical fading, through which the ethical dimensions of a decision fade from view (Tenbrunsel & Messick, 2004). Cognitive dissonance is reduced through euphemistic relabeling of illicit practices, as shown in financial-crime narratives (Cahyono et al., 2025). The normalization of corruption describes how such justifications become institutionalized and socialized within organizations (Ashforth & Anand, 2003), and research on everyday dishonesty shows that people cheat to the extent that they can preserve a positive self-concept (Mazar et al., 2008). The concept of bounded ethicality, which describes systematic and largely unconscious limits on people’s ability to recognize the ethical implications of their own decisions (Chugh et al., 2005), connects these processes to the heuristics-and-biases tradition: the same cognitive constraints that distort risk judgments also limit moral awareness. This suggests that risk-based and moral distortions are not separate families of mechanisms but two expressions of bounded cognition operating on the same decision.

### 4.5. Implications for Prevention and Compliance

The findings also carry important implications for prevention. Traditional deterrence models assume that increasing sanction severity or detection probability will reduce misconduct (Becker, 1968). However, if overconfidence reduces perceived sanction likelihood, then deterrence mechanisms may be cognitively discounted.

Similarly, if neutralization techniques (Sykes & Matza, 1957) and moral disengagement (Bandura, 1999) reduce anticipatory guilt, compliance programs focused solely on rule dissemination may be insufficient. Psychologically informed interventions should directly address rationalization strategies and moral reframing processes.

Governance reforms such as separating CEO and chair roles may function as structural correctives to overconfidence-driven dominance. Strengthening ethical culture may disrupt social learning processes consistent with differential association principles (Sutherland, 1949). These findings suggest that effective prevention requires aligning structural controls with an understanding of cognitive vulnerabilities.

Translating these findings into guidelines for managers and compliance officers requires distinguishing interventions supported by the reviewed studies from those extrapolated from the broader behavioral-ethics literature. Interventions with direct support in the corpus are mainly structural: board independence and the separation of CEO and chair roles, which constrain the expression of executive overconfidence; external monitoring through analyst coverage, short selling and audit quality, which raises perceived detection probability; and compensation structures that reduce extreme short-term performance pressure. These strategies are summarized in Table 4.

A second group of interventions is theoretically consistent with the identified mechanisms but has been tested mainly outside white-collar settings. (1) Structured decision protocols for high-risk decisions, such as revenue recognition or related-party transactions, requiring documented consideration of alternatives and of how a regulator would judge the decision, to counter overconfidence and motivated reasoning. (2) Pre-mortem and adversarial red-team reviews of major financial reports and strategic decisions, in which a designated team argues that misconduct or misstatement has occurred, to counter the illusion of control. (3) Ethics training that teaches employees to recognize neutralization phrases and euphemistic framing in their own and others’ reasoning, rather than merely disseminating rules, to target rationalization. (4) Moral reminders and commitment signatures placed at the point of decision, which reduce dishonesty by making self-concept maintenance more difficult (Mazar et al., 2008). (5) Protected whistleblowing channels combined with clear individual accountability, to counter the diffusion and displacement of responsibility. (6) Rotation of auditors and key control personnel, together with monitoring of team-level norms, to interrupt social learning and the normalization of deviance. Managers should treat this second group as hypotheses to be evaluated in their own settings rather than as established remedies.

### 4.6. Cognitive Biases and Dispositional Profiles: Dark Personality, Corporate Psychopathy and Type A Behavior

Dispositional traits are, together with moral self-regulation failures, the mechanisms examined most often in the included studies, and the behavioral repertoires associated with situational cognitive distortions resemble those described for dispositional profiles. Corporate psychopathy has been associated with bullying, unfair supervision and counterproductive work behavior (Boddy, 2011), and psychopathic traits may be masked by charisma and impression management in managerial settings (Babiak et al., 2010). Overconfidence, entitlement-based rationalization and the dehumanization of stakeholders involved in moral disengagement overlap with the grandiosity, callousness and lack of remorse that characterize psychopathy and, more broadly, the dark triad (Paulhus & Williams, 2002). The Type A pattern of competitiveness, time urgency and hostility (Friedman & Rosenman, 1974) likewise resembles the performance-pressure profile identified in Table 1, Table 2 and Table 3. Because later research attributed much of the predictive value of the Type A pattern to its hostility component rather than to the global pattern (Chida & Steptoe, 2009), we treat it here as a hypothesized correlate centred on competitiveness and hostility rather than as a validated offender profile.

Two interpretations are compatible with the evidence. Under an interaction model, dispositional traits increase susceptibility to specific biases—individuals high in psychopathy or narcissism, for example, may be more prone to overconfidence and moral disengagement (Egan et al., 2015)—so that biases mediate or moderate the effect of personality on misconduct. Under an independence model, biases operate in otherwise ordinary professionals, as the slippery-slope and self-concept maintenance literatures suggest (Campana, 2016; Mazar et al., 2008), and dispositional profiles constitute a separate and smaller pathway. Dispositional traits are the primary mechanism in 33 of the 107 included studies, 26 of them added through the updated searches, but few studies examine traits and situational cognitive biases within the same design, so the question cannot yet be resolved.

The evidence on traits is also not uniform: in one comparison of imprisoned fraudsters with community controls, the fraudsters reported lower psychopathy and less non-prosocial emotional manipulation than the controls (Wang et al., 2021), which illustrates the limits of self-report measures in offender samples. The overlap between traits and biases nonetheless matters for practice. Workplace bullying, mobbing and other hostile managerial behaviors are observable in a way that cognitive distortions are not, and they may therefore serve as early warning signals, or red flags, of a leadership profile in which cognitive distortions and dispositional traits converge. Linking harassment-reporting channels with fraud-risk assessment and internal audit would allow such signals to inform situational prevention before misconduct escalates. This link has not been tested in the included studies and is proposed as a direction for future research and practice.

### 4.7. Cross-Cultural Validity of Cognitive Bias Measures

The geographic concentration documented in Section 3.2 raises a measurement question in addition to a sampling one. Most operationalizations of cognitive biases in the corpus were developed in the United States, Western Europe or China, and many rely on proxies tied to specific institutional settings, such as executives’ option-exercise behavior or media portrayals of CEOs. Whether these measures capture the same construct across cultures is uncertain. Overconfidence may be expressed differently in individualist and collectivist cultures, where norms of self-enhancement differ (Hofstede, 2001); rationalization may draw on different neutralization techniques where loyalty to the group or family carries greater moral weight; and the deterrent meaning of detection probability differs between common-law systems with active private enforcement and civil-law systems that rely more heavily on public enforcement (La Porta et al., 1998). Proxy-based measures are particularly vulnerable, because the behaviors they rely on depend on compensation practices and market regulation that vary across countries. Future research should test the measurement invariance of psychometric bias instruments across cultural groups and validate archival proxies against direct measures before comparing effects across countries, especially in Latin America, Africa and the Middle East, where evidence is scarce.

### 4.8. Knowledge Gaps and Research Agenda

Table 5 organizes the main knowledge gaps identified by this review around the three theoretical traditions introduced in Section 2.1, together with two cross-cutting themes. Three priorities stand out. First, the criminological tradition needs direct measures of perceived sanction certainty among actual or potential offenders, so that the effect of overconfidence on perceived risk is tested rather than inferred from archival proxies. Second, the neutralization and moral-disengagement tradition needs longitudinal and field designs that capture how justifications develop over the course of a fraud, since most current evidence is cross-sectional or retrospective. Third, the organizational-wrongdoing tradition needs multilevel designs that measure cognition at the individual, team and organizational levels simultaneously. Across traditions, the most consequential gap is integrative: few studies test, within the same sample, how risk-based and moral distortions combine and how organizational context conditions their joint effect.

### 4.9. Contribution to Criminal Psychology

This review contributes to criminal psychology in three principal ways. First, it consolidates fragmented empirical evidence into a coherent synthesis of cognitive mechanisms underlying white-collar crime. Second, it integrates behavioral decision theory with established criminological frameworks, including the Fraud Triangle (Cressey, 1953) and neutralization theory (Sykes & Matza, 1957). Third, it advances a multilevel cognitive–organizational model that highlights the interaction between psychological distortions and structural contexts.

By demonstrating that corporate misconduct frequently emerges from systematic distortions in risk perception, moral reasoning, and motivational processing, this study supports a psychologically grounded understanding of white-collar crime that complements traditional structural models.

These interpretations should nonetheless be read alongside the methodological limitations detailed in Section 2.7—most notably the reliance on English-language search terms, the retrospective registration of the original review stages, the moderate size of the corpus and probable publication bias toward significant findings—which constrain the strength and generalizability of the cognitive–organizational model proposed here.

## 5. Conclusions

This systematic review examined the role of cognitive biases and psychological mechanisms in white-collar, corporate, and financial misconduct. By synthesizing 107 studies—empirical and conceptual—published between 1975 and 2026, the review provides an integrated account of how distortions in perception, judgment, and moral reasoning contribute to organizational deviance.

The findings show that two families of mechanisms dominate the literature: moral self-regulation failures, particularly rationalization, neutralization and moral disengagement, and dispositional traits, particularly psychopathy, narcissism and the dark triad. Motivational pressures and risk-perception distortions such as overconfidence are examined less often, mainly in archival studies of corporate fraud. These biases operate through identifiable proximal processes, including risk underestimation, guilt reduction, ethical detachment, and motivational pressure internalization. Importantly, their criminogenic impact is not autonomous but contingent upon organizational and institutional environments. Weak governance structures, permissive cultures, and inadequate monitoring systems amplify the behavioral consequences of cognitive distortions, whereas strong oversight mechanisms attenuate them.

The review advances white-collar crime scholarship by integrating behavioral decision-making theory with established criminological frameworks. Specifically, it demonstrates how cognitive biases function as psychological catalysts within the Fraud Triangle dimensions of pressure, opportunity, and rationalization. In doing so, it reframes the Fraud Triangle as a psychologically mediated process rather than a purely structural model.

From a criminal psychology perspective, the findings challenge explanations that rely solely on stable criminal dispositions or rational utility maximization. Instead, misconduct often emerges from predictable cognitive vulnerabilities embedded in routine professional decision-making. This insight shifts the analytical focus from “who the offender is” to “how decisions are cognitively constructed” within organizational settings.

The study also carries practical implications. Prevention strategies must be bias-sensitive rather than generic. Structural reforms that counteract overconfidence, ethics programs that explicitly address neutralization techniques, and cultural interventions that disrupt social learning mechanisms represent psychologically informed approaches to compliance and governance. Designing organizational systems that anticipate and mitigate cognitive distortions may be more effective than relying exclusively on deterrence-based enforcement.

Despite methodological limitations related to publication bias and measurement and methodological heterogeneity, this review contributes to cumulative knowledge development within criminal psychology by consolidating fragmented evidence into a coherent framework. Future research should expand cross-cultural examination, employ direct psychological measurement of cognitive biases, and develop multilevel models that integrate individual cognition with organizational context, measure cognitive biases and dispositional traits jointly, and incorporate Spanish- and Portuguese-language scholarship from Latin America.

Finally, white-collar crime is not only a structural or economic phenomenon but also a cognitive one. Understanding how biased perception, moral reframing, and motivational distortions shape executive decision-making is essential for advancing both theory and prevention. By placing cognitive mechanisms at the center of analysis, this study offers a more psychologically grounded account of corporate misconduct and provides a foundation for future interdisciplinary inquiry.

## Figures and Tables

**Table 1:** Most Frequently Examined Cognitive Biases in White-Collar Crime.

Cognitive Bias Category	Specific Bias/Mechanism	Primary Psychological Function	Types of Misconduct Associated	Predominant Level of Analysis	Representative Empirical Evidence
Overconfidence Bias	Overconfidence, hubris, illusion of control	Underestimation of risk; overestimation of ability	Corporate fraud, financial misreporting, money laundering	CEO/TMT	(Cao et al., 2024; Saeidi, 2022; Zhong et al., 2023)
Rationalization Mechanisms	Neutralization, moral justification, euphemistic framing	Reduction in guilt; preservation of moral self-image	Fraud, corruption, laundering	Individual/Group	(Cahyono et al., 2025; Maulidi & Muftah Ali, 2025)
Moral Disengagement	Diffusion of responsibility, displacement of blame	Detachment from ethical standards	Greenwashing, corporate misconduct	Individual/Organizational	(He et al., 2022; Egan et al., 2015)
Performance Pressure Bias	Fear of falling, underperformance pressure	Heightened perceived necessity to commit misconduct	Fraud escalation, price-fixing	Executive/Organizational	(Zhong et al., 2023; Piquero, 2012)
Escalation of Commitment	Sunk cost fallacy, persistence bias	Continued engagement in failing or fraudulent strategies	Long-term fraud schemes	Executive	(Campana, 2016)
Social Learning Bias	Normalization of deviance; imitation	Internalization of deviant norms	Executive misconduct	Individual/Contextual	(Kallunki et al., 2026)
Ethical Sensitivity Differences	Gender-based ethical orientation	Enhanced monitoring sensitivity	Earnings management, fraud	Board/TMT	(Xu et al., 2025; Githaiga, 2024)
Dispositional Traits (Dark Personality)	Psychopathy, narcissism, Machiavellianism, dark triad, low self-control	Reduced empathy and remorse; entitlement; manipulativeness; greater susceptibility to moral disengagement	Corporate fraud, financial crime, bank fraud, acceptance of white-collar crime	Individual/CEO	(Blickle et al., 2006; Ragatz et al., 2012; Lingnau et al., 2017)

Source: self-made, based on the thematic coding of the included studies.

**Table 2:** Interaction Between Cognitive Biases and Organizational/Situational Factors.

Cognitive Bias	Organizational Moderator	Situational Moderator	Type of Interaction	Observed Effect on Misconduct
Overconfidence	Weak governance; low board independence	Policy uncertainty; economic downturn	Amplification	Increased fraud likelihood
Overconfidence	Strong analyst coverage; short selling; audit quality	Market transparency	Attenuation	Reduced fraud or earlier detection
Rationalization	Permissive organizational culture	Group normalization	Reinforcement	Increased co-offending
Moral Disengagement	Weak internal controls	Environmental regulatory pressure	Mediation	Increased greenwashing
Performance Pressure Bias	High variable compensation	Financial underperformance	Mediation	Increased fraud incentives
Social Learning	Lack of ethical culture	High-misconduct local environments	Contextual amplification	Higher executive misconduct
Gender Ethical Sensitivity	Board diversity	Gender-egalitarian institutional norms	Conditional moderation	Reduced fraud (context-dependent)

Source: self-made.

**Table 3:** Theoretical Pathways Linking Cognitive Distortions to Criminal Outcomes.

Cognitive Bias	Proximal Psychological Process	Fraud Triangle Component Activated	Mechanism Type	Criminal Outcome
Overconfidence	Risk underestimation	Opportunity	Cognitive distortion	Corporate fraud commission
Overconfidence	Detection underestimation	Opportunity	Perception bias	Reduced detection probability
Rationalization	Guilt reduction; moral reframing	Rationalization	Moral disengagement	Recurrent misconduct
Moral Disengagement	Dehumanization of stakeholders	Rationalization	Ethical detachment	Greenwashing
Underperformance Bias	Financial pressure internalization	Pressure	Motivational distortion	Fraud escalation
Escalation of Commitment	Persistence despite warning signals	Pressure + Rationalization	Behavioral inertia	Prolonged fraud schemes
Social Learning	Imitation and norm internalization	Opportunity (social)	Normative conformity	Co-offending
Weak Monitoring Context	Reduced perceived sanction certainty	Opportunity	Structural enabler	Increased misconduct

Source: self-made.

**Table 4:** Implications for Prevention, Compliance Design, and Future Research.

Targeted Cognitive Bias	Prevention Strategy	Level of Intervention	Evidence-Based Rationale	Practical Application
Overconfidence	Separate CEO/Chair roles; enhance board independence	Corporate governance	Reduces unchecked executive dominance	Governance reform
Overconfidence	Increase analyst monitoring and market transparency	Institutional	Improves external oversight	Regulatory transparency
Rationalization	Ethics training addressing neutralization techniques	Individual/Organizational	Weakens moral justifications	Behavioral ethics programs
Moral Disengagement	Accountability frameworks; whistleblowing systems	Organizational	Reinforces moral responsibility	Compliance infrastructure
Performance Pressure Bias	Redesign incentive compensation structures	Governance	Reduces excessive short-term pressure	Compensation reform
Social Learning Bias	Strengthen ethical culture and mentoring	Organizational	Disrupts deviant norm transmission	Culture-building programs
Detection Bias	Independent auditing; partial observability models	Institutional	Improves detection and deterrence	Audit and enforcement reform
Institutional Norms	Promote inclusive governance norms	Macro-level	Strengthens diversity-based fraud mitigation	Policy reform

Source: self-made.

**Table 5:** Knowledge gaps and research agenda by theoretical tradition.

Theoretical Tradition	Knowledge Gap	Priority Research Questions	Suggested Designs	Level of Analysis
Criminological/rational choice	Biases inferred from archival proxies; perceived sanction certainty rarely measured	Does overconfidence reduce perceived detection probability among managers? Do deterrence signals lose effect among overconfident executives?	Scenario experiments with managers; surveys linked to archival data	Individual
Neutralization and moral disengagement	Mostly cross-sectional or retrospective evidence; few studies of actual offenders	How do justifications develop over the course of a fraud? Which neutralizations precede, and which follow, offending?	Longitudinal designs; interviews with convicted offenders; analysis of court records and internal communications	Individual/Group
Organizational wrongdoing	Cognition rarely measured above the individual level	Do individual biases aggregate into an organizational cognitive climate, by composition or by compilation?	Multilevel and multi-source designs; network analysis of co-offending	Team/Organization
Dispositional profiles (cross-cutting)	Biases and dark traits or Type A patterns rarely measured together	Are cognitive biases mediators, moderators or independent of dark-triad and Type A effects? Is workplace bullying an early signal?	Joint measurement of traits and biases; bullying records linked to misconduct	Individual
Integration across traditions	Risk-based and moral distortions studied in separate clusters	How do overconfidence and rationalization combine within the same decision?	Process-tracing experiments; mixed-methods case studies	Individual/Organization
Context and culture	Evidence concentrated in the US, Europe and East Asia; measures not tested for invariance	Are bias measures invariant across cultures and legal systems? Do mechanisms differ in Latin America?	Cross-national comparisons; measurement-invariance testing; Spanish- and Portuguese-language research	Country/Institution

Source: self-made.

## Data Availability

The full data-extraction spreadsheet, the coding protocol, and the Bibliometrix (R) script used for the bibliometric analyses are available from the corresponding author upon reasonable request.

## Supplementary Materials

The following supporting information can be downloaded at: https://www.mdpi.com/article/10.3390/bs16101915/s1, Table S1: Included studies (n = 107), with design category, coding and quality appraisal (MMAT 2018 for empirical studies; JBI Checklist for Text and Opinion for conceptual studies); Table S2: Search strategies and records excluded at the full-text stage, with reasons; Table S3: Coding of psychological mechanisms in the 107 included studies; File S1: PRISMA 2020 Checklist.
