# Supplementary material for: Cognitive Biases and Moral-Regulatory Mechanisms in White-Collar Crime: A Systematic Review and Bibliometric Mapping of Psychological Pathways to Corporate and Financial Misconduct
Source: Behav Sci (Basel). 2026 Oct 15;16(10):1915. doi: 10.3390/bs16101915 (PMC13701460; doi:10.3390/bs16101915)
Supplement: Supplementary file 1 [file behavsci-16-01915-s001.zip › PRISMA_2020_checklist_behavsci-4590941_1709.pdf]

PRISMA 2020 Checklist — behavsci-4590941

Cognitive Biases and Moral-Regulatory Mechanisms in White-Collar Crime: A Systematic Review and Bibliometric Mapping of Psychological Pathways to Corporate and Financial Misconduct

Legend: green = reported and complete yellow = partially reported / justified absence, minor adjustment red = not reported, needs new text before submitting the checklist

Section and Topic	Item #	Checklist item	Location where item is reported / Status
**TITLE**			
Title	1	Identify the report as a systematic review.	Title, p.1 — the title already contains "A Systematic Review of Psychological Mechanisms...". Complete.
**ABSTRACT**			
Abstract	2	See the PRISMA 2020 for Abstracts checklist.	Abstract, p.1. Verified against MDPI's Behavioral Sciences author instructions: this journal does NOT require a structured abstract with headings (unlike some sister MDPI journals, e.g., Brain Sciences). Unstructured format is acceptable as-is. Complete.
**INTRODUCTION**			
Rationale	3	Describe the rationale for the review in the context of existing knowledge.	Section 1, Introduction, paragraphs 4–6 (p.2–3): fragmentation of the literature and absence of a comprehensive synthesis. Complete.
Objectives	4	Provide an explicit statement of the objective(s) or question(s) the review addresses.	Section 1, final paragraph (p.3): four explicit objectives (i–iv). Complete.
**METHODS**			
Eligibility criteria	5	Specify the inclusion and exclusion criteria for the review and how studies were grouped for the syntheses.	RESOLVED. Section 2.3 lists explicit inclusion/exclusion criteria. Criterion 1 was broadened to accept empirical research OR a substantive conceptual/theoretical contribution, after abstract-level review found ~10 of the 71 included studies are conceptual/review pieces rather than primary empirical studies; corpus-description language throughout the manuscript (abstract, 2.1, 2.4, 3.4, Conclusions) was updated accordingly to say "71 studies" rather than "71 empirical studies." Complete.
Information sources	6	Specify all databases, registers, websites, organisations, reference lists and other sources searched or consulted to identify studies. Specify the date when each source was last searched or consulted.	RESOLVED. Section 2.2: WoS Core Collection identified as sole source. Search date confirmed as 5 May 2026 (raw export of the 71 included records verified against this date; a supplementary re-run on 15 Sept 2026 found no additional eligible records). Add "(searched 5 May 2026)" to Section 2.2 text.
Search strategy	7	Present the full search strategies for all databases, registers and websites, including any filters and limits used.	Section 2.2, p.5–6: full Boolean search string reported verbatim. Complete.
Selection process	8	Specify the methods used to decide whether a study met the inclusion criteria of the review, including how many reviewers screened each record and each report retrieved, whether they worked independently, and if applicable, details of automation tools used in the process.	RESOLVED. New paragraph added to Section 2.3: four reviewers (J.D.-P., N.L.-S., P.S.-G., O.G.-H.) with complementary disciplinary expertise; disagreements resolved by group discussion and consensus. Complete.
Data collection process	9	Specify the methods used to collect data from reports, including how many reviewers collected data from each report, whether they worked independently, any processes for obtaining or confirming data from study investigators, and if applicable, details of automation tools used in the process.	RESOLVED. New sentence added to Section 2.4: data extraction conducted by the same four reviewers as selection, collaboratively, disagreements resolved by consensus. Complete.
Data items	10a	List and define all outcomes for which data were sought. Specify whether all results that were compatible with each outcome domain in each study were sought, and if not, the methods used to decide which results to collect.	PARTIAL. Section 2.4 lists coded variables (bias type, mechanism, moderators, framework) but the review is conceptual/thematic rather than outcome-based; clarify explicitly that no outcome-level effect data were sought (state as N/A with justification).
Data items	10b	List and define all other variables for which data were sought (e.g. participant and intervention characteristics, funding sources). Describe any assumptions made about any missing or unclear information.	Section 2.4, p.6–7: publication year, country, method, level of analysis, misconduct type, theoretical framework, moderators. Missing: how unclear/missing data were handled — add one sentence.
Study risk of bias assessment	11	Specify the methods used to assess risk of bias in the included studies, including details of the tool(s) used, how many reviewers assessed each study and whether they worked independently, and if applicable, details of automation tools used in the process.	RESOLVED (as a justified absence). New paragraph added at the start of Section 2.6: explains that no formal RoB tool was applied given the thematic/conceptual synthesis design, and names three

Section and Topic	Item #	Checklist item	Location where item is reported / Status
			indirect quality safeguards (WoS peer-review threshold; 4-reviewer screening; coded methodological-approach variable). Declared as a limitation. Acceptable for this review design.
Effect measures	12	Specify for each outcome the effect measure(s) (e.g. risk ratio, mean difference) used in the synthesis or presentation of results.	RESOLVED. New consolidated paragraph added at the end of Section 2.5: explicit "Not applicable" statement — no effect measures were defined or pooled, consistent with the thematic/bibliometric (non-meta-analytic) design.
Synthesis methods	13a	Describe the processes used to decide which studies were eligible for each synthesis.	RESOLVED. New paragraph in Section 2.5 explicitly states that eligible studies were grouped for synthesis according to the categories defined in Section 2.4, consistent with the eligibility criteria in Section 2.3.
Synthesis methods	13b	Describe any methods required to prepare the data for presentation or synthesis, such as handling of missing summary statistics, or data conversions.	RESOLVED. Covered by the new consolidated "Not applicable" paragraph in Section 2.5 (no summary statistics were prepared/converted for pooling).
Synthesis methods	13c	Describe any methods used to tabulate or visually display results of individual studies and syntheses.	Section 2.5 + Tables 1–4 (Section 3). Complete.
Synthesis methods	13d	Describe any methods used to synthesize results and provide a rationale for the choice(s). If meta-analysis was performed, describe the model(s), method(s) to identify statistical heterogeneity, and software package(s) used.	RESOLVED. New sentence in Section 2.5 clarifies thematic coding was performed using a shared spreadsheet; Bibliometrix (R) is specified for the bibliometric mapping. Rationale for the synthesis choice (thematic + bibliometric, no meta-analysis) is given.
Synthesis methods	13e	Describe any methods used to explore possible causes of heterogeneity among study results (e.g. subgroup analysis, meta-regression).	RESOLVED (labeled). Covered by the new consolidated "Not applicable" paragraph in Section 2.5; the qualitative counterpart (contextual moderators) remains discussed narratively in Section 3–4.
Synthesis methods	13f	Describe any sensitivity analyses conducted to assess robustness of the synthesized results.	RESOLVED. Covered by the new consolidated "Not applicable" paragraph in Section 2.5.
Reporting bias assessment	14	Describe any methods used to assess risk of bias due to missing results in a synthesis (arising from reporting biases).	RESOLVED. New consolidated paragraph in Section 2.5 explicitly states no formal reporting-bias assessment (e.g., funnel plot, Egger's test) was performed, as this requires a quantitative synthesis the design does not produce. Section 2.6 separately retains the narrative discussion of publication-bias risk as a limitation.
Certainty assessment	15	Describe any methods used to assess certainty (or confidence) in the body of evidence for an outcome.	RESOLVED. Covered by the new consolidated "Not applicable" paragraph in Section 2.5.
**RESULTS**			
Study selection	16a	Describe the results of the search and selection process, from the number of records identified in the search to the number of studies included in the review, ideally using a flow diagram.	Section 2.3, p.7 + Figure 1 (PRISMA flow diagram). Complete.
Study selection	16b	Cite studies that might appear to meet the inclusion criteria, but which were excluded, and explain why they were excluded.	PARTIAL (transparently declared, not resolvable). The itemized list of the 64 full-text exclusions was never preserved and cannot be reconstructed. Section 2.6 now explicitly discloses this as a transparency limitation, per PRISMA guidance to state when such information is unavailable. Aggregate counts and general exclusion criteria remain reported in 2.3.
Study characteristics	17	Cite each included study and present its characteristics.	PARTIAL — RESOLVED for bibliographic fields. Supplementary Table S1 built from the verified WoS export: lists all 71 included studies (authors, title, source, year, document type, WoS category, DOI). Four coding columns (bias/mechanism, level of analysis, country, theoretical framework) are still blank pending transfer from the authors' original coding spreadsheet. Also resolve 3 missing DOIs (Lederman 2021; Willison & Lowry 2018; Haugh 2017) before submission.
Risk of bias in studies	18	Present assessments of risk of bias for each included study.	RESOLVED (as justified absence). New sentence at the start of Section 3.1 explicitly notes that no study-level risk-of-bias ratings are presented, consistent with Section 2.5, and points readers to Supplementary Table

Section and Topic	Item #	Checklist item	Location where item is reported / Status
			S1 for descriptive methodological characteristics instead. Complete, tied to item 11.
Results of individual studies	19	For all outcomes, present, for each study: summary statistics and an effect estimate and its precision.	RESOLVED. Covered by the new consolidated "Not applicable" paragraph in Section 2.5.
Results of syntheses	20a	For each synthesis, briefly summarise the characteristics and risk of bias among contributing studies.	PARTIAL. Section 3 and Tables 1–4 summarise characteristics by theme; risk-of-bias integration is missing (tied to item 11/18).
Results of syntheses	20b	Present results of all statistical syntheses conducted, incl. heterogeneity, if meta-analysis was done.	RESOLVED. Covered by the new consolidated "Not applicable" paragraph in Section 2.5.
Results of syntheses	20c	Present results of all investigations of possible causes of heterogeneity among study results.	RESOLVED (labeled). Covered by the new consolidated statement in Section 2.5; qualitative equivalent (contextual moderators) remains in Section 3–4.
Results of syntheses	20d	Present results of all sensitivity analyses conducted.	RESOLVED. Covered by the new consolidated "Not applicable" paragraph in Section 2.5.
Reporting biases	21	Present assessments of risk of bias due to missing results for each synthesis assessed.	RESOLVED. Covered by the new consolidated statement in Section 2.5 (tied to item 14).
Certainty of evidence	22	Present assessments of certainty (or confidence) in the body of evidence for each outcome assessed.	RESOLVED. Covered by the new consolidated "Not applicable" paragraph in Section 2.5 (tied to item 15).
**DISCUSSION**			
Discussion	23a	Provide a general interpretation of the results in the context of other evidence.	Section 4 (4.1–4.6), p.20–24. Complete.
Discussion	23b	Discuss any limitations of the evidence included in the review.	RESOLVED. New paragraph added at the end of Section 4 (before Conclusions) explicitly cross-references the Section 2.6 limitations (single database, no formal RoB tool, publication-bias risk) in the interpretive context PRISMA expects.
Discussion	23c	Discuss any limitations of the review processes used.	RESOLVED. Same new cross-reference paragraph at the end of Section 4 covers review-process limitations (single-database search).
Discussion	23d	Discuss implications of the results for practice, policy, and future research.	Section 4.5 ("Implications for prevention and compliance") and Section 5 (Conclusions), future-research directions. Complete.
**OTHER INFORMATION**			
Registration and protocol	24a	Provide registration information for the review, including register name and registration number, or state that the review was not registered.	RESOLVED. Registered on protocols.io. New sentence added to Section 2.1 and a "Registration:" line added to the back matter, both citing DOI: https://doi.org/10.17504/protocols.io.5qpvoj7bbg4o/v2. Complete.
Registration and protocol	24b	Indicate where the review protocol can be accessed, or state that a protocol was not prepared.	RESOLVED. The full protocol (10 steps: objectives, rationale, eligibility, sources, search strategy, selection, extraction, risk of bias, synthesis, registration disclosure) is publicly accessible at the DOI above. Complete.
Registration and protocol	24c	Describe and explain any amendments to information provided at registration or in the protocol.	RESOLVED (as N/A). No amendments: the protocol was registered retrospectively, after the review was already complete, so there is no prior registered version to amend. This is explicitly disclosed in the registration itself (Step 10: Registration Status Disclosure) and in the manuscript. Complete.
Support	25	Describe sources of financial or non-financial support for the review, and the role of the funders or sponsors in the review.	Back matter: "Funding: This research received no external funding." Complete.
Competing interests	26	Declare any competing interests of review authors.	Back matter: "Conflicts of Interest: The authors declare no conflicts of interest." Complete.
Availability of data, code and other materials	27	Report which of the following are publicly available and where they can be found: template data collection forms; data extracted from included studies; data used for all analyses; analytic code; any other materials used in the review.	RESOLVED. Back matter now specifies: Supplementary Table S1 (extracted data on the 71 included studies); the full data-extraction spreadsheet, coding protocol, and Bibliometrix (R) script available from the corresponding author on reasonable request. Complete.

From: Page MJ, McKenzie JE, Bossuyt PM, Boutron I, Hoffmann TC, Mulrow CD, et al. The PRISMA 2020 statement: an updated guideline for reporting systematic reviews. BMJ 2021;372:n71. doi: 10.1136/bmj.n71.
